# Recurrent repeat expansions in human cancer genomes

**DOI:** 10.1038/s41586-022-05515-1

**Published:** 2022-12-14

**Authors:** Graham S. Erwin, Gamze Gürsoy, Rashid Al-Abri, Ashwini Suriyaprakash, Egor Dolzhenko, Kevin Zhu, Christian R. Hoerner, Shannon M. White, Lucia Ramirez, Ananya Vadlakonda, Alekhya Vadlakonda, Konor von Kraut, Julia Park, Charlotte M. Brannon, Daniel A. Sumano, Raushun A. Kirtikar, Alicia A. Erwin, Thomas J. Metzner, Ryan K. C. Yuen, Alice C. Fan, John T. Leppert, Michael A. Eberle, Mark Gerstein, Michael P. Snyder

**Affiliations:** 1 Department of Genetics, Stanford University, Stanford, CA USA; 2 Department of Biomedical Informatics, Columbia University, New York, NY USA; 3 New York Genome Center, New York, NY USA; 4 Illumina, Inc., San Diego, CA USA; 5 Division of Oncology, Department of Medicine, Stanford University School of Medicine, Stanford, CA USA; 6 Data Science Program, Northwestern University, Chicago, IL USA; 7 Genetics and Genome Biology, The Hospital for Sick Children, Toronto, Ontario Canada; 8 Department of Molecular Genetics, University of Toronto, Toronto, Ontario Canada; 9 Department of Urology, Stanford University School of Medicine, Stanford, CA USA; 10 Veterans Affairs Palo Alto Health Care System, Palo Alto, CA USA; 11 Division of Nephrology, Department of Medicine, Stanford University School of Medicine, Stanford, CA USA; 12 Computational Biology and Bioinformatics Program, Yale University, New Haven, CT USA; 13 Molecular Biophysics and Biochemistry Department, Yale University, New Haven, CT USA; 14 Department of Computer Science, Yale University, New Haven, CT USA

**Keywords:** Genome, Genetic variation, Cancer genomics

## Abstract

Expansion of a single repetitive DNA sequence, termed a tandem repeat (TR), is known to cause more than 50 diseases^1,2^. However, repeat expansions are often not explored beyond neurological and neurodegenerative disorders. In some cancers, mutations accumulate in short tracts of TRs, a phenomenon termed microsatellite instability; however, larger repeat expansions have not been systematically analysed in cancer^3–8^. Here we identified TR expansions in 2,622 cancer genomes spanning 29 cancer types. In seven cancer types, we found 160 recurrent repeat expansions (rREs), most of which (155/160) were subtype specific. We found that rREs were non-uniformly distributed in the genome with enrichment near candidate *cis*-regulatory elements, suggesting a potential role in gene regulation. One rRE, a GAAA-repeat expansion, located near a regulatory element in the first intron of *UGT2B7* was detected in 34% of renal cell carcinoma samples and was validated by long-read DNA sequencing. Moreover, in preliminary experiments, treating cells that harbour this rRE with a GAAA-targeting molecule led to a dose-dependent decrease in cell proliferation. Overall, our results suggest that rREs may be an important but unexplored source of genetic variation in human cancer, and we provide a comprehensive catalogue for further study.

## Abstract (web summary)

An atlas explores the landscape of recurrent repeat expansions in human cancer genomes.

## Main

Expansions of tandem DNA repeats (TRs) are known to cause more than 50 devastating human diseases, including Huntington’s disease and fragile X syndrome^1,2^. TR tracts that cause human disease are typically large (more than 100 bp)^1^. However, identifying large TRs with short-read DNA sequencing methods is difficult because the repeat sequences are ubiquitous in the genome and many are too large—larger than the typical sequencing read length—to uniquely map to the reference genome^9^. Thus, many large TRs go undetected with current genomic technologies, and, despite their importance to monogenic disease, the frequency and function of recurrent repeat expansions (rREs) are unknown in complex human genetic diseases such as cancer^10^.

Previous studies have profiled the landscape of alterations in short TRs (STRs) in cancer genomes^3–5^. In particular, microsatellite instability (MSI)^6–8^, defined by an alteration in the lengths of STRs, is prevalent in various types of cancer, including in endometrial (30%), stomach (20%) and colorectal (15%) cancers^3,4,11–13^. However, systematic analysis of the frequency of genome-wide large TR expansions has not been studied in cancer even though such expansions were posited to exist more than 25 years ago^14^.

Recently, new bioinformatic tools to identify repeat expansions in short-read whole-genome sequencing (WGS) datasets^15–18^ have led to the identification of both known and novel repeat expansions in human disease, primarily in the area of neurological disorders where repeat expansions have historically been studied^15–23^. Here we analysed 2,622 human cancer genomes with matching normal samples for the presence of somatic repeat expansions. We identified 160 recurrent repeat expansions (rREs) in seven types of cancer, including many rREs located in or near known regulatory elements. One of these rREs was observed in 34% of kidney cancers, and targeting this repeat expansion with sequence-specific DNA binders led to a dose-dependent decrease in cellular proliferation. Overall, our approach identifies a new class of recurrent changes in cancer genomes and provides an initial resource of these changes.

## Recurrent repeat expansions

**Extended Data Fig. 1:**
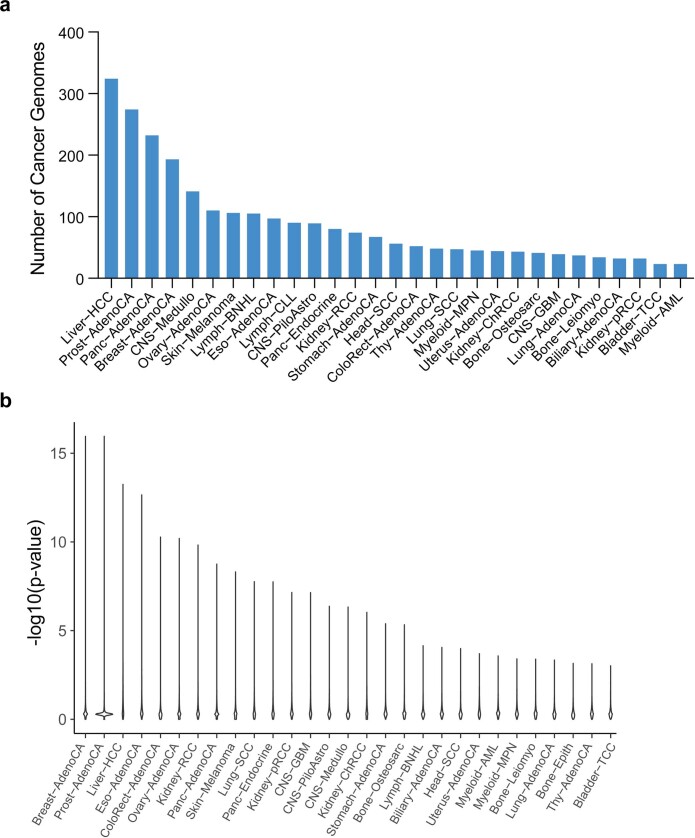
Overview of PCAWG data and analysis with ExpansionHunter De Novo. **a**, Distribution of cancer genomes analysed across 29 human cancers in the PCAWG data. **b**, Distribution of p-values following candidate recurrent repeat expansion (rRE) analysis with ExpansionHunter Denovo (one-sided Wilcoxon rank-sum test).

We collected uniformly processed alignments of WGS data for tumour–normal pairs in the International Cancer Genome Consortium (ICGC) and The Cancer Genome Atlas (TCGA), both a part of the Pan-Cancer Analysis of Whole Genomes (PCAWG) datasets^24^. After filtering, these data consisted of 2,622 cancer genomes from 2,509 patients across 29 different cancer types (Extended Data Fig. 1). Each cancer type was treated as its own cohort and was analysed independently of the other cancer types. We called somatic rREs with ExpansionHunter Denovo (EHdn) (Methods), which measures TRs whose length exceeds the sequencing read length in short-read sequencing datasets^25,26^. That is, EHdn performs case–control comparisons using a non-parametric statistical test to determine whether repeat length is longer in tumour genomes than in matching normal genomes. This approach is analogous to joint population-level genotyping.

**Extended Data Fig. 2:**
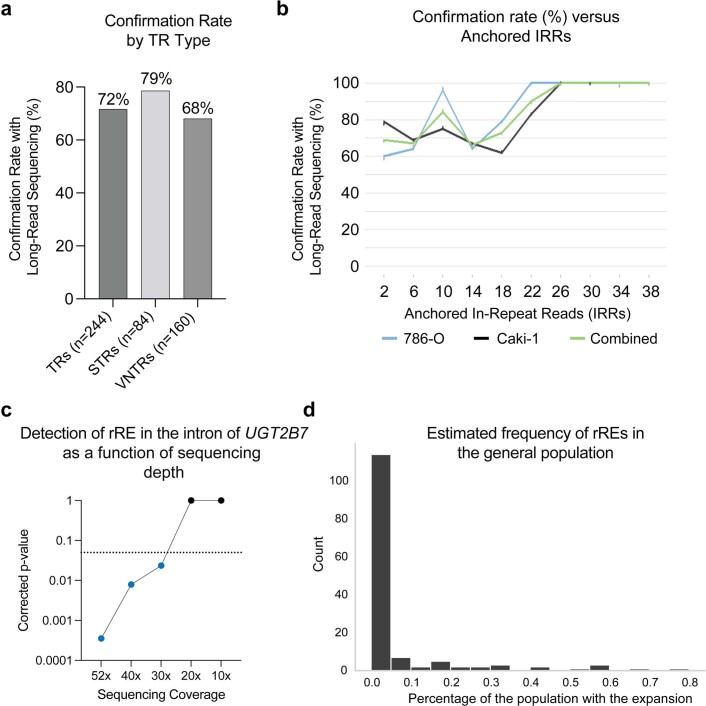
Benchmarking EHdn. **a**, Comparison of anchored in-repeat reads (IRRs) to long-read sequencing reads. Long-read sequencing confirmation rate across all tandem repeats (TRs, motifs 2–20 bp), short TRs with motifs from 2–6 bp, and variable number TRs with motifs from 7–20 bp. **b**, Confirmation rate versus number of anchored IRRs. **c**, Effect of downsampling on the identification of the rRE in the intron of *UGT2B7* in kidney cancer. Tumour genomes from the PCAWG dataset were downsampled to the specified number. ExpansionHunter De Novo was run, and the resulting Bonferroni-correct p-value is depicted for the given sequencing coverage. Corrected p-value from one-sided Wilcoxon rank-sum test with Bonferroni correction. **d**, Estimation of the frequency of repeat expansions in rRE loci in the general population. The number of rREs (count) corresponding to each bin is plotted on the y-axis. Results are from analysis of 1000 Genomes Project samples^60^ (n = 2,504) (GRCh38) and Medical Genome Reference Bank^61^ samples (n = 4,010).

We first confirmed the accuracy of EHdn by performing whole-genome short- and long-read sequencing on the 786-O and Caki-1 cancer cell lines. We found that EHdn captured 72% of the repeat expansions observed in long-read sequencing (Extended Data Fig. 2). We also tested the effect of sequencing coverage on the detection of rREs and found that EHdn was robust down to 30× coverage (Extended Data Fig. 2). We then analysed 2,622 matching tumour and normal genomes with EHdn (285,363 TRs). We identified 578 candidate rREs (locus-level false discovery rate (FDR) < 10%).

**Extended Data Fig. 3:**
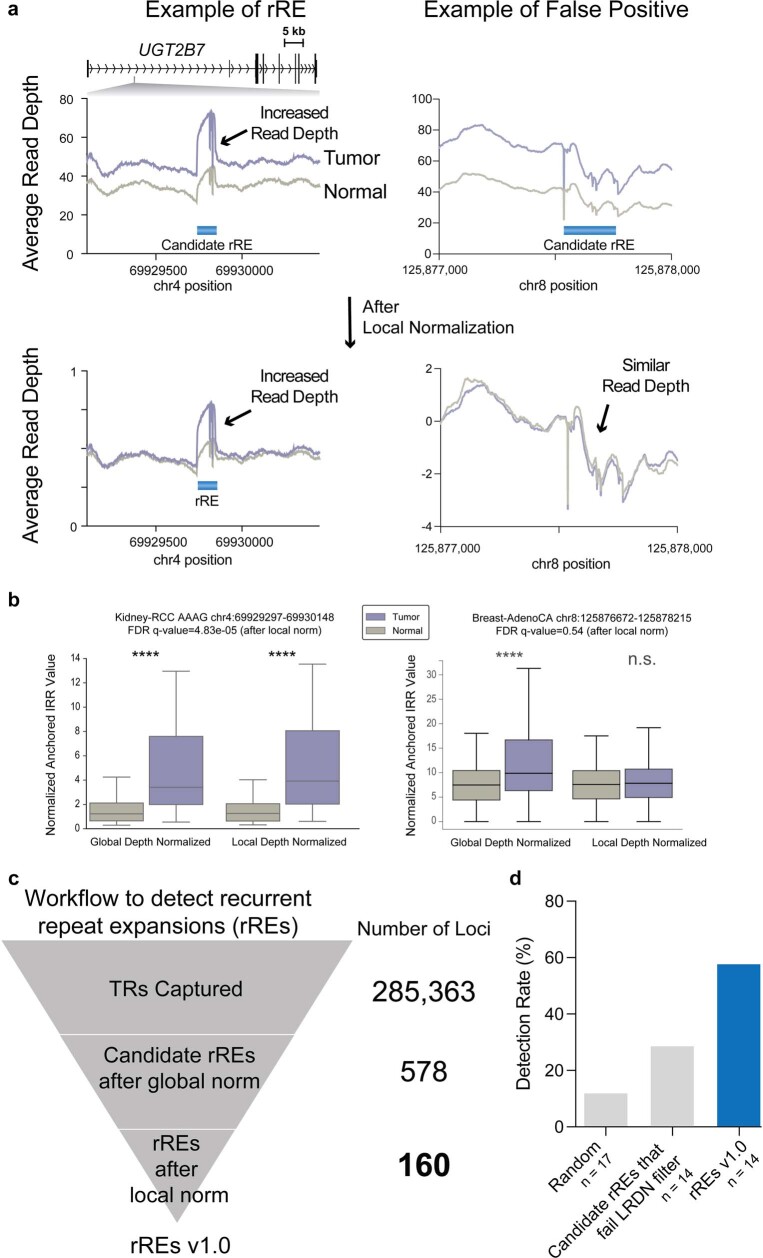
Local read depth normalization of recurrent repeat expansion (rRE) candidates. **a**, Examples of read depth before and after local normalization. **b**, Examples of anchored in-repeat read (IRRs) before and after local normalization. The read depth for the locus on the left is derived from TCGA data, and the read depth for the locus on the right is derived from PCAWG data. Q-values were calculated from two-tailed Student’s t-test with FDR correction by Benjamini-Hochberg. FDR q-value=4.83e-05 and 0.54 for Kidney-RCC and Breast-AdenoCA, respectively (*n* = 74 Kidney-RCC genomes and *n* = 193 Breast-AdenoCA genomes analyzed). **c**, Workflow to identify rREs. **d**, Detection rate in an independent cohort of samples.

EHdn is expected to be sensitive to the copy number variations observed in cancer genomes. To account for copy number variants, we devised and implemented a local read depth filtering method that normalizes the signal originating from repeat reads using the read depth in the vicinity of the TR (Methods and Extended Data Fig. 3). We benchmarked the local read depth normalization approach with simulated chromosomal amplifications ranging from two (diploid) to ten copies. We found that this filter accounted for changes in chromosomal copy number in a manner superior to standard global read depth normalization (Extended Data Fig. 3). Overall, we conclude that local read depth normalization is valuable to identify bona fide rREs in cancer genomes and that many of the rREs that pass the filter are expanded in cancer. For example, without local read depth normalization, we could detect only 31% of candidate rREs in independent cohorts of matching tumour–normal tissue samples for breast, prostate and kidney cancers (15, 18 and 12 patients, respectively). Our local read depth filtering approach removed more than 75% (418/578) of false-positive candidate rREs (Extended Data Fig. 3). Notably, several rRE candidates that were removed are situated in hotspots for chromosomal amplification, such as chromosomal 8q amplifications that increase *MYC* production in breast cancer (Extended Data Fig. 3)^27^. Our analysis suggests that the standalone EHdn method may have selected these loci owing to amplification rather than repeat expansions, and their removal is thus important.

After implementing our local read depth filtering strategy, we increased our detection rate to 57% (8/14) in independent cohorts (Extended Data Fig. 3). Notably, the loci we could not validate had lower expansion frequencies (5–12%). These rREs may be real but may also have been more difficult to validate in the small validation cohorts (Supplementary Table 6). Thus, we believe that this number may be an underestimate of the independent detection rate. Of the 14 candidate rREs that failed our local read depth filter, 29% (4/14) were detected in independent cohorts of samples, indicating that the filtering removes most loci that cannot be validated (Extended Data Fig. 3), but removes some true positives as well.

**Fig. 1:**
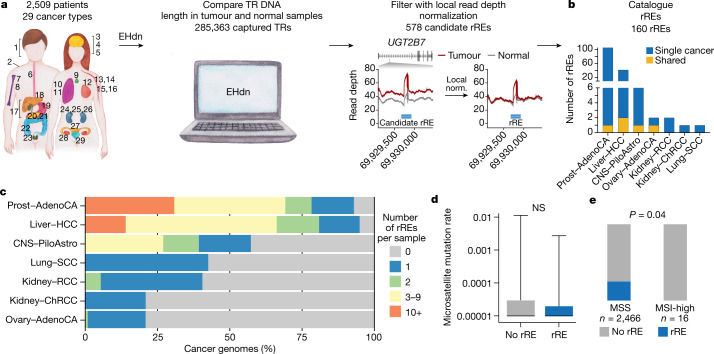
Genome-wide detection of rREs in cancer genomes. **a**, Scheme of the method to identify rREs in 2,509 patients across 29 human cancer types: 1, head and neck squamous cell carcinoma (Head−SCC); 2, skin–melanoma; 3, glioblastoma (CNS–GBM); 4, medulloblastoma (CNS−Medullo); 5, pilocytic astrocytoma (CNS–PiloAstro); 6, oesophageal adenocarcinoma (Oeso−AdenoCA); 7, osteosarcoma (Bone−Osteosarc); 8, leiomyosarcoma (Bone−Leiomyo); 9, thyroid adenocarcinoma (Thy–AdenoCA); 10, lung adenocarcinoma (Lung−AdenoCA); 11, lung squamous cell carcinoma (Lung−SCC); 12, mammary gland adenocarcinoma (Breast−AdenoCA); 13, B cell non-Hodgkin lymphoma (Lymph−BNHL); 14, chronic lymphocytic leukaemia (Lymph−CLL); 15, acute myeloid leukaemia (Myeloid−AML); 16, myeloproliferative neoplasm (Myeloid−MPN); 17, biliary adenocarcinoma (Biliary–AdenoCA); 18, hepatocellular carcinoma (Liver−HCC); 19, stomach adenocarcinoma (Stomach−AdenoCA); 20, pancreatic adenocarcinoma (Panc−AdenoCA); 21, pancreatic neuroendocrine tumour (Panc−Endocrine); 22, colorectal adenocarcinoma (ColoRect–AdenoCA); 23, prostatic adenocarcinoma (Prost−AdenoCA); 24, chromophobe renal cell carcinoma (Kidney–ChRCC); 25, renal cell carcinoma (Kidney–RCC); 26, papillary renal cell carcinoma (Kidney−pRCC); 27, uterine adenocarcinoma (Uterus−AdenoCA); 28, ovarian adenocarcinoma (Ovary−AdenoCA); 29, transitional cell carcinoma of the bladder (Bladder−TCC). **b**, Distribution of rREs across cancer types. **c**, Proportion of cancer genomes with rREs. **d**, STR mutation rate for cancer genomes with and without an rRE. Two-tailed Mann–Whitney test (*n* = 2,465 cancer genomes); NS, not significant. Boxes extend from the 25th percentile to the 75th percentile, the centre line represents the median and whiskers represent minima and maxima. **e**, Distribution of rREs across MSS and MSI-high cancers. Chi-squared (two-tailed) test with Yates’ correction (*n* = 2,482 cancer genomes).

**Extended Data Fig. 4:**
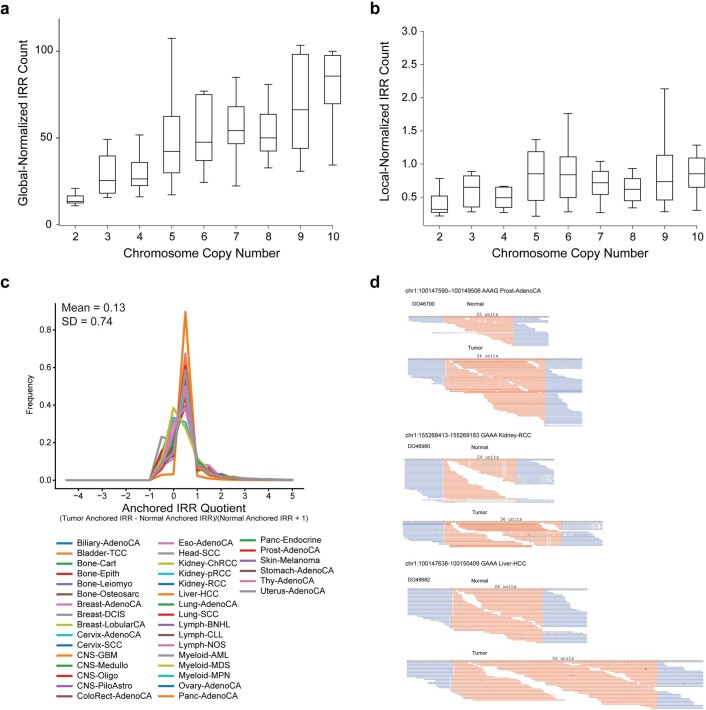
Benchmarking LRDN and EHdn. **a,b**, Benchmarking the local read depth normalization filter (*n* = 10 loci analysed). **c**, The anchored IRR quotient was calculated as (tumour anchored IRR – normal anchored IRR)/(normal anchored IRR + 1). Dashed line at 2.5 indicates the threshold for calling a locus as a repeat expansion in a cancer genome. **d**, ExpansionHunter was used to estimate repeat sizes from short-read sequencing data, and the results were visualized with REViewer (see Methods). The allele with the longest repeat tract for normal and tumour samples is shown. The TR is depicted in red, and the flanking regions are depicted in blue.

After accounting for local read depth, we detected 160 rREs in seven human cancer types (rRE catalogue v1.0; Fig. 1). We expected high concordance with ExpansionHunter given that this tool is related to EHdn, and indeed we observed 91% concordance with ExpansionHunter (Extended Data Fig. 4). We found that most (80%) of these loci were rarely expanded in the general population (<5% of the time, *n* = 6,514 genomes; Extended Data Fig. 2). rREs were primarily observed in prostate and liver cancers, but we also detected rREs in ovarian, pilocytic astrocytoma, renal cell carcinoma (RCC), chromophobe RCC and squamous cell lung carcinoma. Thus, rREs are found in tissues derived from each of the three primary germ layers (ectoderm, mesoderm and endoderm), suggesting that these expansions are a phenomenon inherent to the human genome rather than any tissue-specific process. We next performed a preliminary analysis to estimate the presence of somatic repeat expansions in individual cancer genomes. In prostate and liver cancers, most cancer genomes (93% and 95%, respectively) contained at least one rRE, with some genomes harbouring several rREs (Fig. 1c). For some pathogenic repeats, a larger TR length at birth predisposes an individual to somatic repeat expansions later in life^1,2^, but we did not generally observe this with rREs (Supplementary Table 7). Overall, rREs were found in 7 of the 29 human cancer types examined and were largely cancer subtype specific.

**Extended Data Fig. 5:**
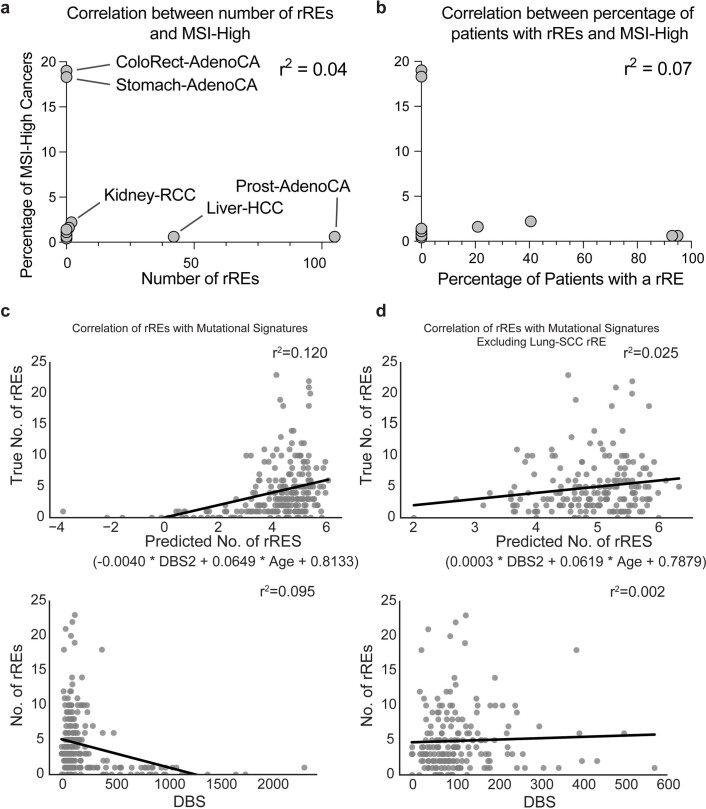
Association of rREs with genetic features. **a**, Correlation of rREs with MSI-High cancers.** b**, **c**, Association of rREs with mutational signatures. **b**, Correlation between DBS2 and the number of rREs detected. **c**, Correlation between DBS2 and the number of rREs detected when Lung-SCC data are omitted from the analysis.

We next examined whether rREs correlate with changes in MSI^3,4^. We determined whether samples harbouring an rRE had a higher mutation rate in STRs, which is a hallmark of MSI^3,28^. We did not observe any significant difference in STR mutation rate for genomes with an rRE compared with those lacking an rRE (two-tailed Wilcoxon rank-sum test, *P* = 0.27; Fig. 1d). We also compared cancer genomes harbouring rREs with cancer genomes previously identified as MSI, using recent results from the PCAWG consortium^28^. We did not observe any enrichment in MSI for samples harbouring an rRE and instead found a weak but significant preference for rREs in microsatellite-stable (MSS) samples, not MSI samples (two-tailed Wilcoxon rank-sum test, *P* = 0.04; Fig. 1e and Extended Data Fig. 5). Thus, our findings might suggest a model in which rREs are formed by a process that is distinct from MSI.

In addition to MSI, different mutational processes lead to a signature of somatic mutations. We tested whether rREs are associated with known mutational signatures by comparing them to 49 signatures of single-base substitutions (SBS) and 11 signatures of doublet-base substitutions (DBS)^29^. We performed multiple linear regression to predict the number of rREs in a sample on the basis of SBS and DBS signatures. Only one DBS signature, DBS2, showed a very weak association with rREs (*r*^2^ = 0.12) (Extended Data Fig. 5).

## Some rREs overlap regulatory elements

**Fig. 2:**
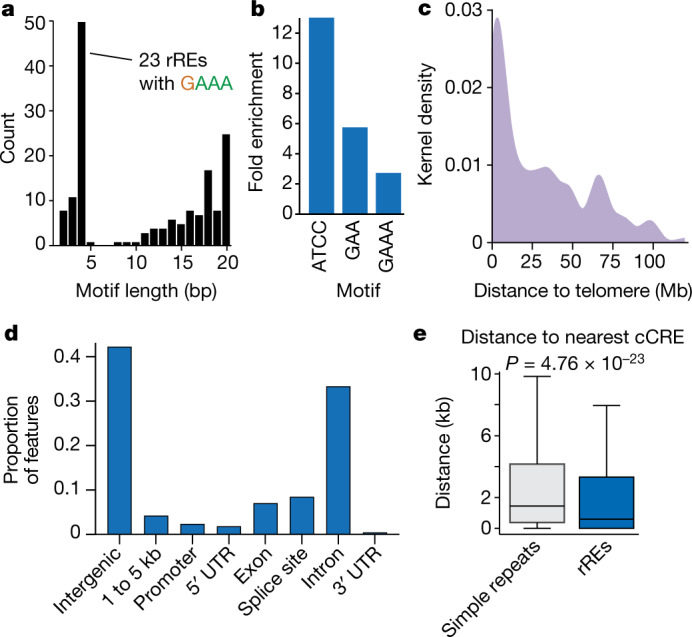
Features of rREs. **a**, Distribution of the repeat unit (motif) for rREs. **b**, Motifs enriched in the catalogue of rREs. **c**, Distance of rREs to the end of the chromosome arm. **d**, Proportion of genic features that overlap with rREs. **e**, Distance of simple repeats (*n* = 950,091 loci) and rREs (*n* = 160 loci) to the nearest Encyclopedia of DNA Elements (ENCODE) cCRE. Centre values represent the median. Welch’s *t* test (two tailed).

**Extended Data Fig. 6:**
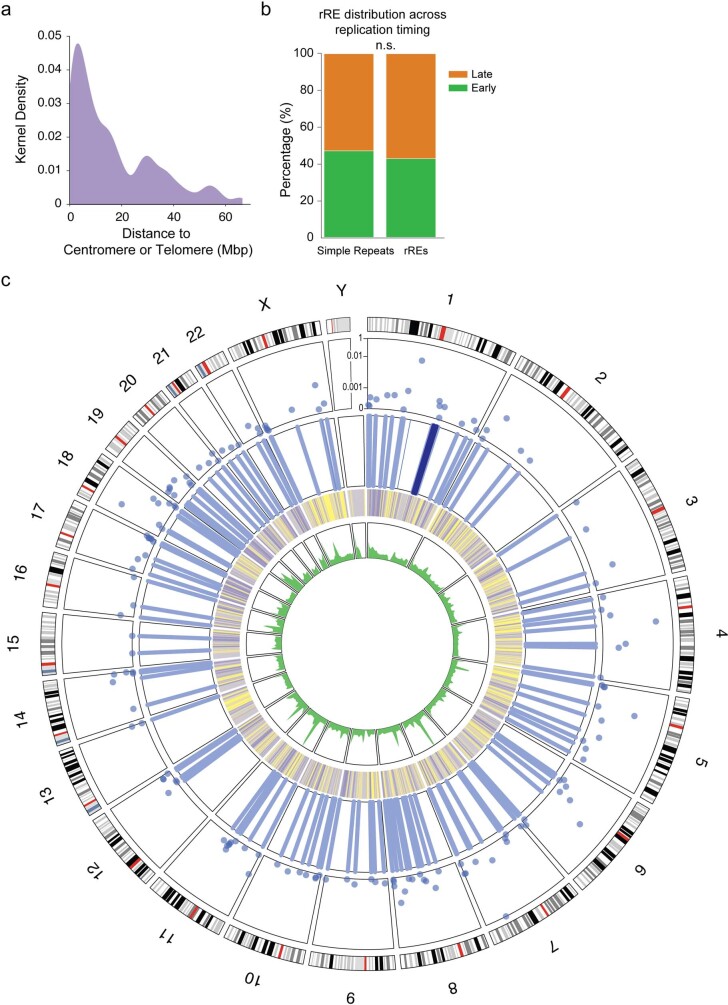
Distribution of rREs across the genome. **a**, Distance of rREs to the nearest centromere or telomere. **b**, Distribution of rREs across early- and late-replicating regions of the genome. Welch’s *t*-test (two-tailed, not significant). **c**, Circos plot depicting (from outside to inside) p-value of rREs, location of rREs where darker shading indicates the rRE observed across 3 cancers, early and late replicating regions (yellow and purple, respectively), and simple sequence repeats. This plot depicts the overlay between different data types and the distribution of rREs across the genome.

**Extended Data Fig. 7:**
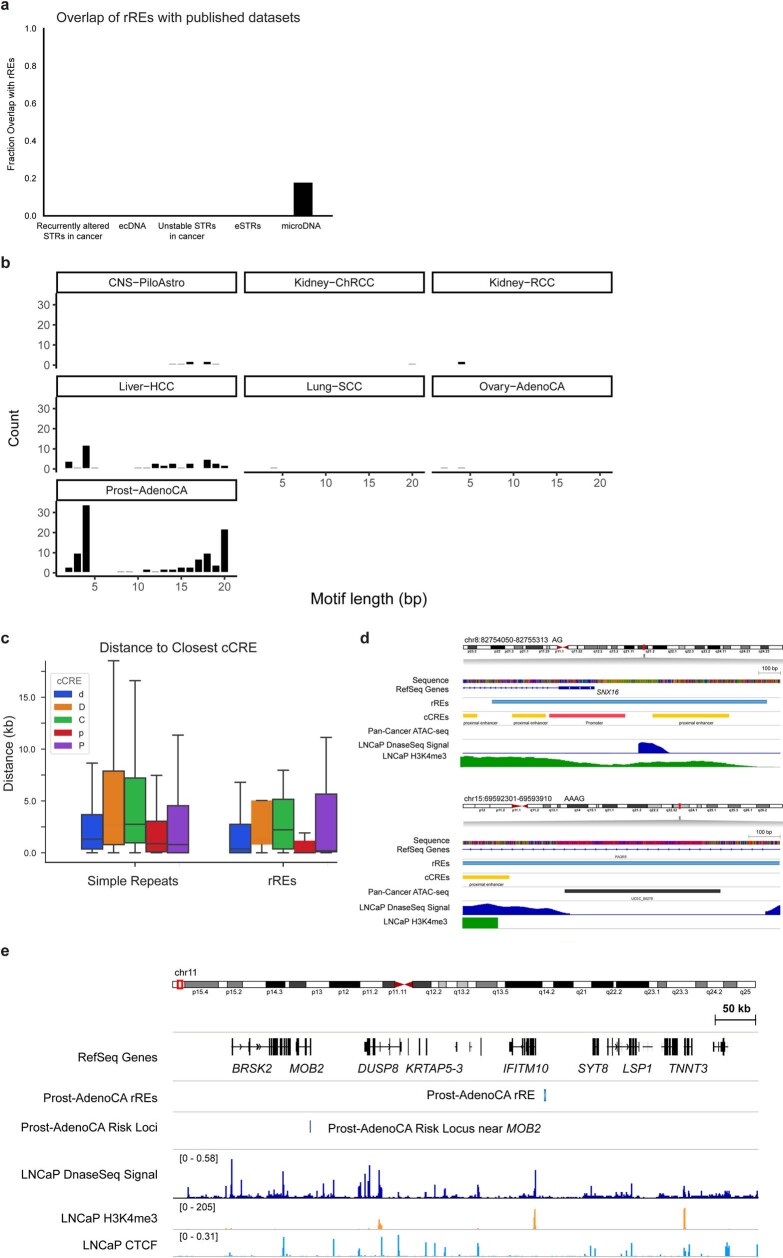
Molecular features of rREs. **a**, Overlap of rREs with other datasets. The fraction of rREs overlapping with other catalogues of TRs and genomic instability. From left to right in the figure, recurrently altered STRs in cancer (Supplementary Data 14 from ref. ^4^; PMID: 28585546), extrachromosomal circular DNA (ecDNA, circular amplification events from Supplementary Table 1; ref. ^62^; PMID: 31748743), unstable STRs in cancer (Supplementary Table 10 from ref. ^3^; PMID: 27694933), eSTRs (Supplementary Data 1; ref. ^63^; PMID: 31676866), and microDNA (From C4-2, ES2, LNCaP, OVCAR8, and PC-3 cells; ref. ^64^; PMID: 26051933). The PubMed ID for each corresponding manuscript is included in the figure. For the overlap of rREs with microDNA, we looked at loci that we attempted to detect in an independent cohort of cancer samples, and we found that we tested 11 loci. Of the 11 rREs tested, 8 (72%) were detected in the independent cohort of cancer samples. **b**, Distribution of rRE motif length across cancer types. **b**,**c**, Association of rREs with regulatory elements. **b**, Distance of simple sequence repeats and rREs to the nearest candidate cis-regulatory elements (cCREs). Key: promoter-like signature (P), proximal enhancer-like signature (p), distal enhancer-like signature (d), DNase-H3K4me3 (D), and CTCF-only (C). **c**, Signal tracks depicting rREs near regulatory elements (*n* = 950,091 simple repeats and *n* = 160 rREs). **d**, Association between rREs in prostate cancer and risk loci in prostate cancer. Signal trace showing an rRE detected in prostate cancer and a risk locus for prostate cancer.

Among the 160 rREs, we observed a variety of different motifs (Supplementary Table 1) whose repeat unit length followed a bimodal distribution, in line with REs identified in other diseases (Fig. 2a and Extended Data Figs. 6 and 7)^26^. rREs were distributed across a range of G+C content, and approximately half (76/160) had a G+C content of less than 50% (Supplementary Table 1). Six rREs contained a known pathogenic motif, all of which were GAA^30^. We examined whether any motifs were enriched in the rRE catalogue as compared with the Tandem Repeat Finder (TRF) catalogue. Although this enrichment could arise from a biological and/or technical process, we found that one of the three enriched motifs was GAA (Fig. 2b). As an example, Friedreich’s ataxia is caused by a repeat expansion of a GAA motif in the intron of the gene encoding frataxin. This expansion results in DNA methylation and the deposition of repressive chromatin marks, leading to robust repression of the gene and development of disease^30^. Because of this, we suspect that some of the rREs found in cancer might alter the epigenome and affect gene regulatory networks.

rREs were distributed non-uniformly across the genome, with a bias towards the ends of chromosome arms (Fig. 2c and Extended Data Fig. 6). This observation is consistent with previous reports of TRs and structural variants^16,31^. We also examined the distribution of rREs relative to gene features with annotatr (Fig. 2d)^32^. The 7% of rREs labelled as exonic appeared proximal to, but not within, exons, but others were in introns, untranslated regions (UTRs) and splice sites. These results suggest that rREs may have different functional roles in the regulation of gene expression.

We measured the distance between rREs and candidate *cis*-regulatory elements (cCREs)^33^; cCREs comprise approximately 1 million functional elements, including promoters, enhancers, DNase-accessible regions and insulators bound by CCCTC-binding factor (CTCF). An rRE near a regulatory element could alter the function of that regulatory element, as is observed in fragile X syndrome and Friedreich’s ataxia^1^. Interestingly, rREs were located closer to cCREs than expected by chance, and we found that 54 of the 160 rREs directly overlapped with a known cCRE (Welch’s *t* test, *P* = 4.76 × 10^–23^; Fig. 2e and Extended Data Fig. 7). Thus, rREs are often found in or near functional regions of the genome.

## rREs with a connection to cancer

**Fig. 3:**
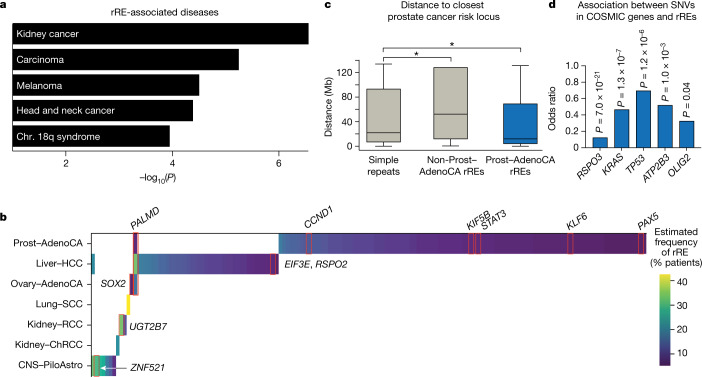
Association of rREs with cancer. **a**, Association of rREs with human diseases. Chr., chromosome. **b**, Estimated frequency of rREs in genes of interest, including nine COSMIC genes. **c**, Distance of simple repeats (*n* =  950,091 loci), non-prostate cancer rREs (*n* = 55 loci) and prostate cancer rREs (*n* = 105 loci) to the nearest prostate cancer risk locus. Centre values represent the median. Statistical significance was measured with Welch’s *t* test (two tailed; *, *q* = 0.08). See Methods section ‘Statistics and reproducibility’ for more information.  **d**, Association between SNVs in genes in the COSMIC tier 1 genes and the presence of rREs. Two-tailed Student’s *t* test with FDR correction by the Benjamini–Hochberg method.

We mapped each rRE to the nearest gene and found that nine rREs mapped to tier 1 genes present in the Catalogue of Somatic Mutations in Cancer (COSMIC) database (Fig. 3 and Supplementary Table 1). We also observed a strong correlation with cancer-related genes (Jensen disease–gene associations^34^). That is, four of the top five diseases associated with the collection of 160 rREs were cancers (Fig. 3b and Supplementary Table 4).

To examine whether some rREs have a role in oncogenesis, we looked at their association with previously identified cancer risk loci. Many rREs were identified in prostate cancer, and 63 loci have previously been associated with susceptibility to prostate cancer from available genome-wide association studies^35^. When we examined the colocalization of rREs and cancer risk loci in prostate cancer, we found that rREs were located closer to prostate cancer susceptibility loci than standard STRs or than would be expected by chance (Student’s *t* test, FDR *q* = 0.08; Fig. 3c and Extended Data Fig. 7).

We next studied the relationship between the occurrence of COSMIC genes and the occurrence of rREs (Fig. 3d). Interestingly, after correcting for multiple-hypothesis testing, somatic mutations were found to occur significantly more in patients’ genomes without rREs for five COSMIC genes. Among these genes, *TP53* in particular is striking, as wild-type *TP53* is critical for mediating the pathogenic effects of repeat expansions in both amyotrophic lateral sclerosis (ALS) and Huntington’s disease^36,37^. In line with these findings, the product of the *RAD53* DNA damage repair gene in yeast is phosphorylated and activated in the presence of an expanded repeat^38^.

**Extended Data Fig. 8:**
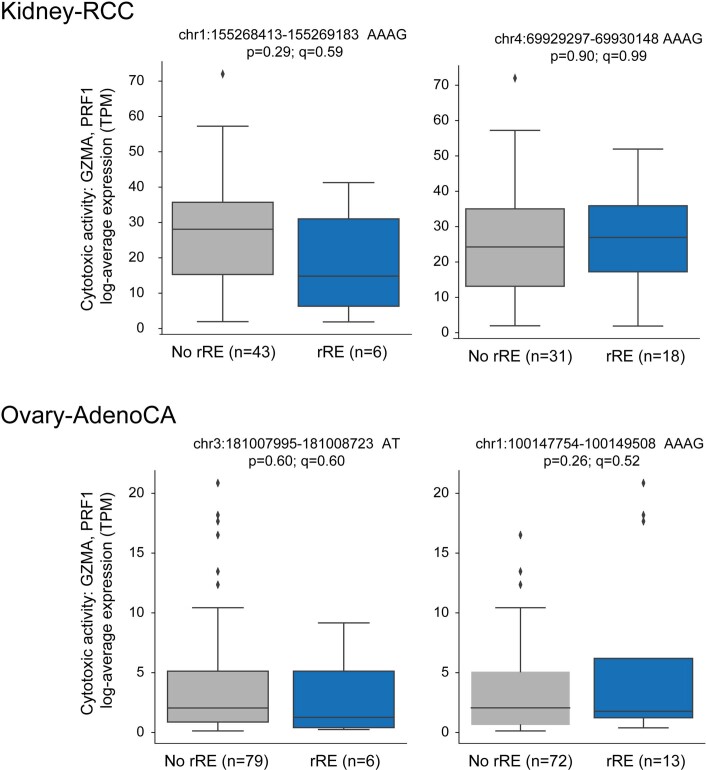
Analysis of cytotoxic activity. **a**, Analysis of *UGT2B7* GAAA rRE in patients with clear cell RCC. N, normal tissue; T, tumour tissue. For gel source data, see Fig. S1. **b**, *UGT2B7* in RCC patients. **b**, Expression of *UGT2B7* (transcripts per million, TPM) in RCC samples as a function of the detection of the rRE in *UGT2B7*. P value computed with Welch’s *t*-test (two-tailed. **c**, Kaplan-Meier survival plots of RCC patients stratified by rRE in the intron of *UGT2B7*. P value computed with Welch’s *t*-test (two-tailed).

MSI-high cancers are often correlated with higher levels of immune cell infiltration^39^. We considered whether some rREs might also be associated with higher immune cell infiltration, but we did not observe a correlation between cytotoxic activity^40^ and the presence of an rRE (Extended Data Fig. 8). Because there were matching RNA sequencing (RNA-seq) data for only 4 of the 160 rREs, this analysis warrants further investigation as more matching WGS and RNA-seq datasets become available.

## An intronic rRE detected in RCC

A GAAA expansion located in the intron of *UGT2B7* was observed in 34% of RCC samples. *UGT2B7* encodes a glucuronidase that clears small molecules—including chemotherapeutics—from the body and is selectively expressed in the kidney and liver^41^.

**Fig. 4:**
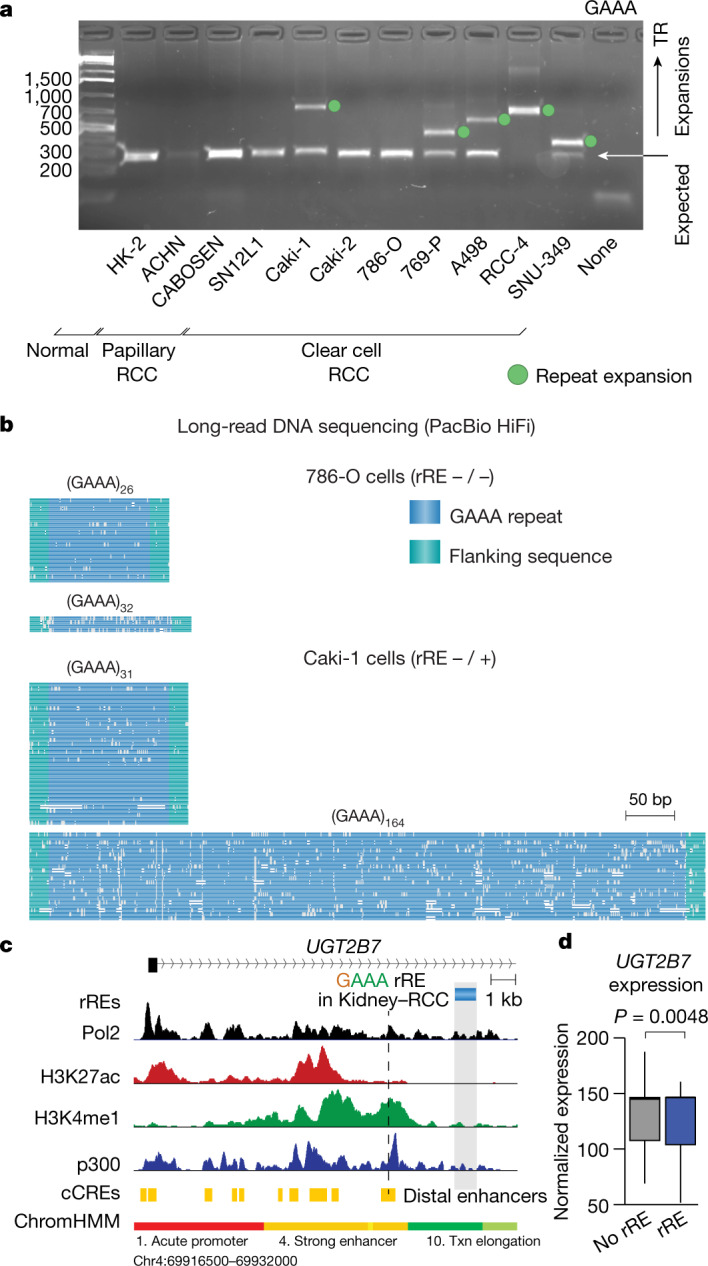
An rRE in RCC. **a**, Gel electrophoresis of the GAAA TR in RCC samples. This analysis was performed in duplicate, and the gel is representative of the results. The units for the ladder are base pairs. For gel source data, see Supplementary Fig. 1. **b**, Visualization of long-read sequencing of the GAAA rRE in the intron of *UGT2B7*. Data are from PacBio HiFi sequencing. **c**, The locus surrounding the rRE detected in the intron of *UGT2B7*. Signal traces of RNA polymerase II (Pol2), acetylated histone H3 lysine 27 (H3K27ac), monomethylated histone H3 lysine 4 (H3K4me1) and p300 in HepG2 cells are shown. cCREs and chromatin states (ChromHMM) are also depicted. Txn, transcription. **d**, Expression of *UGT2B7* isoform ENST00000508661.1 in RCC samples as a function of detection of the rRE in *UGT2B7* (normalized expression, counts). Centre values represent the median. Significance was measured by two-tailed Wald test with FDR correction (Benjamini–Hochberg) (*n* = 49 cancer genomes with matching WGS and RNA-seq data).

**Extended Data Fig. 9:**
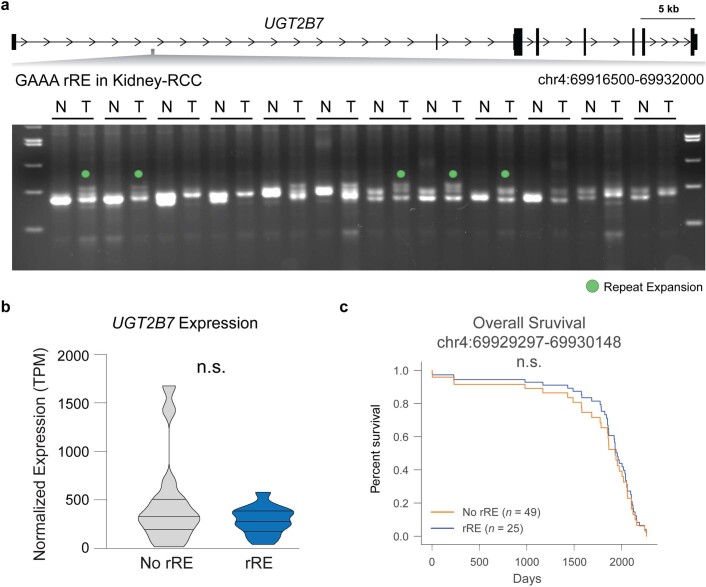
Association of rREs with cytotoxic activity. P values computed with Welch’s *t*-test (two-tailed) with FDR correction (Benjamini-Hochberg) (*n* = 49 Kidney-RCC genomes and *n* = 85 Ovary-AdenoCA genomes analysed).

With gel electrophoresis, we identified the expected TR size of ~26 GAAA repeats in the normal kidney cell line HK-2, corresponding closely to the length observed in the reference genome (Fig. 4a). By contrast, we identified an expansion of between ~63 and ~160 GAAA repeat units in five of eight clear cell RCC cell lines. Most expansions were heterozygous (Fig. 4a). Long-read DNA sequencing with highly accurate PacBio HiFi reads confirmed the PCR results and showed the precise structure of this repeat expansion at single-base-pair resolution for both the 786-O and Caki-1 cell lines (Fig. 4b). We also detected this repeat expansion in 5 of 12 primary kidney tumour tissue samples from patients with clear cell RCC (Extended Data Fig. 9), which showed more heterogeneity than the RCC cell lines; more heterogeneity for human tumour samples than for clonal cell lines might be expected.

Given that *UGT2B7* is selectively expressed in the liver and kidney, and that it has a role in clearing small molecules from the body, we examined whether this rRE may be located near any functional elements that could regulate its expression. Analysis of the chromatin environment surrounding the rRE in *UGT2B7* identified a nearby enhancer, raising the possibility that this rRE alters the expression of *UGT2B7* (Fig. 4c). The repeat motif of this rRE, GAAA, appears similar to the pathogenic repeat motif found in Friedreich’s ataxia, which is GAA. The pathogenic GAA-repeat expansion blocks *FXN* expression^30^. We therefore considered whether the intronic GAAA-repeat expansion might repress the expression of *UGT2B*; we found a modest decrease in expression that was not statistically significant (Extended Data Fig. 8). While this rRE was also not associated with a difference in survival (Extended Data Fig. 8), it was associated with a significant decrease in a transcript isoform of *UGT2B7* (Wald test with FDR correction, *P* = 0.0048) (Fig. 4e). Interestingly, a shift in isoform usage of *UGT2B7* has been noted in cancer^42^.

## Repeat-targeting molecules

**Fig. 5:**
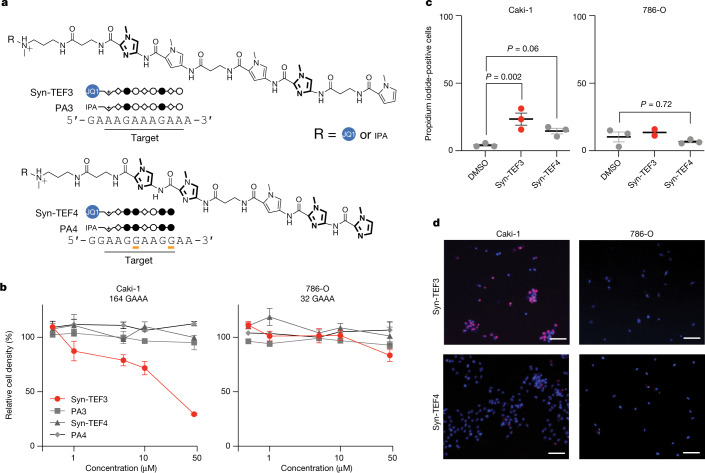
Design and characterization of GAAA-targeting molecules in RCC. **a**, Chemical structures of Syn-TEF3, PA3, Syn-TEF4 and PA4. Syn-TEF3 and PA3 target 5′-AAGAAAGAA-3′. Syn-TEF4 and PA4 target 5′-AAGGAAGG-3′. The structures of *N*-methylpyrrole (open circles), *N*-methylimidazole (filled circles) and β-alanine (diamonds) are shown. *N*-methylimidazole is bolded for clarity. The structure of JQ1 linked to polyethylene glycol (PEG_6_) is represented as a blue circle. The structure of isophthalic acid and its linker is represented as IPA. Complete chemical structures appear in Supplementary Fig. 2. Mismatches formed with Syn-TEF4 and PA4 are indicated with orange lines. **b**, Relative cell density of RCC cell lines Caki-1 and 786-O following treatment (72 h) with compounds as indicated. Relative cell density was measured by CCK-8 assay (Methods). Results are shown as the mean ± s.e.m. (*n* = 4 biological replicates). **c**, Quantification of the percentage of propidium iodide-positive cells. *P* values are from one-way ANOVA with Bonferroni’s correction for multiple comparisons. Results are shown as the mean ± s.e.m. (*n* = 3 biological replicates except *n* = 2 biological replicates for Syn-TEF3 in 786-O cells). **d**, Live-cell microscopy of Caki-1 and 786-O cells stained with propidium iodide (red) and Hoechst 33342 (blue). Scale bars, 100 μm. See also Extended Data Fig. 10.

Do GAAA-repeat expansions contribute to cell proliferation? Targeting pathogenic repeat expansions with small molecules has been demonstrated previously^43^. We previously showed that targeting a related TR motif, GAA, with synthetic transcription elongation factors (Syn-TEF1) reverses pathogenesis in several models of Friedreich’s ataxia^44^. Therefore, if the GAAA rRE in RCC behaves similarly, a Syn-TEF targeting GAAA might have anti-proliferative activity. We rationally designed Syn-TEF3, which contains a GAAA-targeting polyamide and a bromodomain ligand, JQ1, designed to recruit part of the transcriptional machinery (Fig. 5a and Supplementary Fig. 2). We also included a control molecule, Syn-TEF4, which targets GGAA TRs, as well as polyamides PA3 and PA4 that lack the JQ1 domain. We have previously shown that Syn-TEFs and polyamides localize to repetitive TRs in living cells^44,45^.

**Extended Data Fig. 10:**
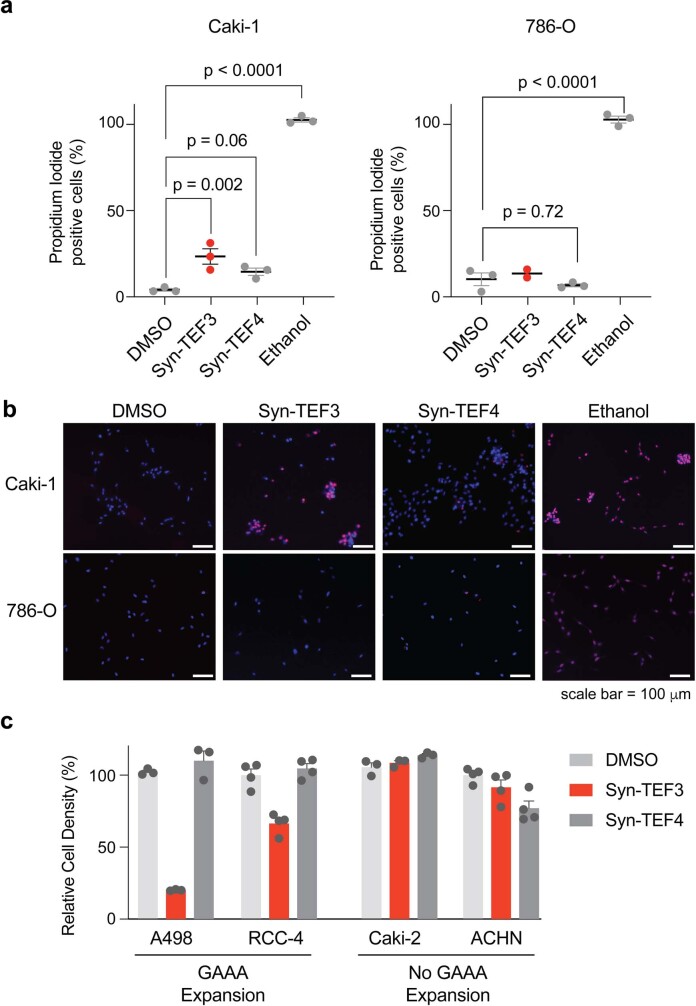
Syn-TEF treatment of RCC cell lines. **a**, Quantitation of the percentage of propidium iodide-positive cells. P values are from a one-way ANOVA adjusted with Bonferroni correction for multiple comparisons. Results are mean ± s.e.m. (*n* = 3 biological replicates, except *n* = 2 biological replicates for Syn-TEF3 in 786-O). **b**, Live cell microscopy of Caki-1 and 786-O cells stained with propidium iodide (red) and Hoechst 33342 (blue). Scale bars, 100 μm. **c**, Relative cell density of RCC cell lines following treatment (72 h) with compounds (50 μM Syn-TEF or 0.1% DMSO vehicle, as indicated). Results are mean ± s.e.m. (ACHN and RCC-4 are *n* = 4 biological replicates, A498 and Caki-2 are *n* = 3 biological replicates).

We examined the effect of Syn-TEFs on cell proliferation (Fig. 5b). Caki-1 and 786-O cells were selected because they have the largest (164 repeats) and smallest (32 repeats) GAAA tracts, respectively, within the first intron of *UGT2B7*. We observed that Syn-TEF3 led to a significant decrease in the proliferation of Caki-1 cells in a dose-dependent manner, but had little effect on 786-O cells. Syn-TEF4, which does not target GAAA TRs, did not significantly decrease proliferation in either of the cell lines tested, demonstrating a requirement for GAAA-specific targeting (Fig. 5b). Two additional cell lines with GAAA-repeat expansions as well as two additional control non-expanded cell lines showed a similar association between Syn-TEF sensitivity and presence of the repeat expansion (Extended Data Fig. 10). In line with this finding, Caki-1 cells treated with Syn-TEF3 exhibited a significant increase in cell death when compared with the DMSO-treated control, as measured by propidium iodide staining (Fig. 5c,d and Extended Data Fig. 10). By contrast, 786-O cells treated with Syn-TEF3 showed no significant difference in propidium iodide-positive cells when compared with DMSO-treated cells (Fig. 5c,d and Extended Data Fig. 10). Notably, the Syn-TEF4, PA3 and PA4 control agents had no significant effect on cell death in either cell line when compared with vehicle control (Fig. 5c,d and Extended Data Fig. 10). These results are preliminary and warrant further study, but they suggest that GAAA-repeat expansions may represent a genetic vulnerability in RCC.

## Discussion

Here we conducted a genome-wide survey of rREs, distinct from MSI, across cancer genomes. Our data (1) identified 160 rREs in seven human cancer types and showed that (2) most (155 of 160) rREs are cancer subtype specific; (3) among diseases, rREs are enriched in human cancer loci and tend to occur near regulatory elements; (4) rREs do not correlate with MSI status; and (5) targeting a GAAA-repeat expansion in RCC with a small molecule leads to cancer cell killing. Taken together, our results uncover an unexplored genetic alteration in cancer genomes with important mechanistic and therapeutic implications.

Cancer cells evolve and adapt in response to environmental or pharmacological perturbations, but the mechanisms supporting these changes are still being uncovered. One source of genetic variation that may enable genetic adaptations is TR DNA sequences. Mutations in the repeat length of TRs can occur up to 10,000 times more frequently than single-nucleotide variants (SNVs) or insertions and deletions (indels)^1^. Repeat expansions may provide a source of genetic variation to enable cancer cells to adapt to changes in the environment^46^. Indeed, colorectal cancers acquire mutations in STRs in response to targeted therapy just 24 h after treatment, suggesting that mutations in these regions may associate with rapid evolution^47^. In future studies, it will be particularly valuable to study repeat expansions in the genomes of cancer cells that face changing environments, including metastasis and chemotherapy.

Historically, MSI has been the focus of efforts to profile changes in STRs in cancer genomes because specific cancer-causing genetic alterations in repair genes can promote widespread STR alterations. Interestingly, we find little to no correlation between rREs and MSI. These results are consistent with previous findings in which the correlation between MSI and repeat instability at larger TRs was not definitive^48^. MSI may contribute to a subtype of rREs that we have not yet uncovered, or rREs may arise from a mutation process that is distinct from that of MSI. There are several different cellular repair systems for DNA, and the rREs we observed are presumably due to very specific locus-associated mechanisms or activities. Some of these repeat expansions may be due to *cis* regions with interesting DNA or chromatin configurations that are prone to expansion at distinct loci, rather than gene mutations that cause global *trans* effects, as occurs in MSI.

There are numerous mechanisms by which a repeat expansion can alter cellular function. Known pathogenic repeat expansions can alter the coding sequence of a protein, such as in the case of Huntington’s disease. However, several repeat expansions in non-coding regions alter gene expression^1^. In other instances, the repeat expansion can lead to a pathogenic RNA molecule (myotonic dystrophy) or protein (ALS)^1^. Finally, repeat expansions in MSI-associated cancers, which are too small to detect by EHdn, can disrupt DNA replication^49^. Thus, our catalogue represents a powerful resource to explore the mechanisms by which rREs alter cellular function in cancer.

Tools to identify repeat expansions are still in their infancy. The field would benefit from cohorts of samples with whole-genome long-read DNA sequencing data, improved bioinformatic methods, increased sequencing coverage and increased cohort sizes. As with other tools that identify repeat expansions from short-read sequencing data, we cannot distinguish zygosity from sample heterogeneity or obtain the precise lengths of repeats. Our independent experimental validation showed that some repeat expansions are heterogeneous (Extended Data Fig. 8). We suspect that tumour heterogeneity may lead to an under-reporting of rREs. Furthermore, this study focuses on somatic mutations, but repeat expansions that occur in the context of normal development will be another important area of study^10^. Furthermore, germline events that predispose an individual to cancer would also be worth studying; there is evidence that a TR in the androgen receptor gene is associated with tumour stage and tumour grade at prostate cancer onset^50^. Finally, we only detected changes in repeat length that were greater than sequencing read length. In future studies, it will be important to explore recurrent changes that are smaller in length. Finally, it is important to acknowledge that rREs could be mediators of phenotypes or passengers that result from genetic instability and clonal selection. In the one instance where we targeted an rRE in RCC, cell proliferation was reduced, in line with a mediator role for this rRE. Distinguishing between these two possibilities for each rRE will be an important line of work in the future.

To our knowledge, this is the first genome-wide survey of repeat expansions beyond a neurological or neurodegenerative disorder. Thousands of high-quality whole-genome sequences exist for many diseases, and our data provide evidence that repeat expansions should be explored beyond the classical bounds of neurodegenerative diseases where they have been most investigated. Our results provide a framework to analyse WGS datasets from complex diseases such as cancer.

## Methods

### Data curation

We obtained white-listed data from the ICGC and TCGA PCAWG dataset. The term ‘white-listed’ refers to samples that passed quality control by the PCAWG consortium^24^. Data were accessed through the Cancer Genome Collaboratory. We used aligned reads (BAM files), which were aligned to GRCh37 as described previously^24^. These data are available through the PCAWG data portal (https://docs.icgc.org/pcawg). A list of samples included in the analysis is available in Supplementary Table 2.

### Identification of somatic rREs

We analysed tumour and matching normal samples for each cancer type independently. We executed EHdn (v0.9.0)^16^ with the following parameters: --min-anchor-mapq 50 --max-irr-mapq 40. To prioritize loci, we developed a workflow termed Tandem Repeat Locus Prioritization in Cancer (TROPIC). We included loci from chromosomes 1–22, X and Y for downstream analysis. We removed loci where >10% of Anchored in-repeat read (IRR) values were >40, which is the theoretical maximum value. The *P* value (from a non-parametric one-sided Wilcoxon rank-sum test) for each locus was used to calculate an FDR *q* value. Loci with FDR < 0.10 are reported. We selected loci where >5% of samples had an Anchored IRR quotient of >2.5. The results of our filtering are available in Supplementary Table 3. For a repeat expansion to be detected by EHdn, the TR was required to be larger than the sequencing read length. A somatic repeat expansion was defined as having FDR *q* < 0.05 in a comparison of the tumour and normal samples. We next calculated a preliminary estimate of the frequency of rREs in each cancer. To call repeat expansions in individual cancer samples, we analysed the distribution of tumour and normal Anchored IRR values and selected a conservative threshold for the Anchored IRR quotient ((tumour Anchored IRR – normal Anchored IRR)/(normal Anchored IRR + 1)) > 2.5 (Extended Data Fig. 4).

### Local read depth normalization

EHdn normalizes the number of Anchored IRRs for a given locus to the global read depth. To account for chromosomal amplifications and other forms of genetic variation that could alter local read depth, we performed the following normalization. For each rRE locus and sample in its corresponding cancer, samtools v1.13 was used with the parameter depth -r to find the read depth at each base pair within the locus and a 500-bp region encompassing the start and stop positions of the TR. We calculated the average read depth at each base pair and defined this as the local read depth. Finally, we calculated the local read depth-normalized Anchored IRR value specific to a sample and rRE combination by dividing the non-normalized Anchored IRR value from EHdn by the local read depth at the locus.

### Generation of CABOSEN cells

CABOSEN cells were generated from a cabozantinib-sensitive (CABOSEN) human papillary RCC xenograft tumour grown in *Rag2*^–/–^*γC*^–/–^ mice, as described previously^51^. Tumour tissue was minced with a sterile blade, and the cell suspension was cultured in DMEM/F-12 medium (Corning) supplemented with 10% (vol/vol) Cosmic calf serum (ThermoFisher). Cells were expanded and cryopreserved in growth medium supplemented with 10% (vol/vol) DMSO, and cells from passage 8 were used for analysis.

### Analysis of rREs by gel electrophoresis

We performed PCR with CloneAmp HiFi PCR Mix (Takara Biosciences) and added DMSO to a final concentration of 5–10% (vol/vol) as needed. A list of the primers used to analyse the loci is available in Supplementary Table 5. All cell lines tested negative for mycoplasma contamination with the MycoAlert Mycoplasma Detection kit (Lonza). Cell line identities were authenticated through STR profiling by the Genetic Resources Core Facility at Johns Hopkins University, with the exception of SNU-349 cells, which did not match the reported STR profile of SNU-349 cells or any other catalogued cell line but had a mutated *VHL* gene and expressed high levels of *PAX8* and *CA9*, in line with a clear cell RCC origin.

### Visualization of repeat expansions with ExpansionHunter and REViewer

To inspect the reads supporting a repeat expansion, we annotated the repeat as described on the GitHub page for ExpansionHunter. We then profiled the region with ExpansionHunter (v4.0.2) using the default settings^15^. The resulting reads were visualized with REViewer (v0.1.1) using the default settings. REViewer is available at https://github.com/Illumina/REViewer. A repeat expansion was called when the repeat tract length for one allele of the tumour sample was greater than 100 bp and exceeded the repeat tract length of both normal alleles. A locus was considered validated if at least ten cancer genomes had a repeat expansion.

### Validation of rREs in independent cohorts of samples

Twelve pairs of matching normal and tumour samples from patients with clear cell RCC were obtained with the patients’ informed consent ex vivo upon surgical tumour resection (Stanford institutional review board-approved protocols 26213 and 12597) and analysed. Eighteen and 15 pairs of matching normal and tumour samples for prostate and breast cancer, respectively, were obtained from the Tissue Procurement Shared Resource facility at the Stanford Cancer Institute and analysed. These samples were obtained with patients’ informed consent (Stanford institutional review board-approved protocols 11977 and 55606). Nucleic acid was isolated with either the Quick Microprep Plus kit (D7005) or the Zymo Quick Miniprep Plus kit (D7003) (Zymo Research). Gel electrophoresis was performed as described above. A locus was considered detected if a somatic repeat expansion was identified in at least one patient tumour sample compared with a matching normal sample.

### Downsampling analysis

For the downsampling analysis, tumour genomes from RCC samples were downsampled from their mean (52×) sequencing depth to 40×, 30×, 20× and 10× depth with the samtools view command. EHdn was run, as described above, for each of the sequencing depths, and the Bonferroni-corrected *P* value was plotted for the rRE in *UGT2B7* (GAAA, chr4:69929297–69930148).

### Benchmarking the local read depth normalization filter

We benchmarked the local read depth filter in silico by observing its behaviour with simulated reads. First, we created a reference genome containing artificially expanded repeats. We randomly selected ten TRs located on chromosome 1 that were shorter than the sequencing read length of 100 bp. We artificially expanded these TRs on chromosome 1 of GRCh37 with the BioPython Python package (v1.79). Next, we used wgsim (v0.3.1-r13) to simulate reads from the reference file with the command ‘wgsim -N 291269925 --1 100 --2 100 reference_file.fasta output.read1.fastq output.read2.fastq’. The number of reads (specified by the -N option) was calculated to achieve 30× coverage of chromosome 1. The resulting pair of files, hereafter referred to as the base fastq files, contained a copy number of 2 for all of the expansions.

To simulate copy number amplification, the read simulation process was repeated using reference files that contained only the artificially expanded repeats and their surrounding 1,000-bp flanking regions. We created ten pairs of fastq files, each with an increasing copy number. We specified the copy number by multiplying the number of reads to generate (wgsim -N option) by the required number. To generate the final set of fastq files, we concatenated each pair of copy number-amplified fastq files with the base fastq files. The end result was eight pairs of fastq files that contained reads for chromosome 1 and copy number amplification varying from 2 to 10 of the expanded repeats.

The base fastq file with a copy number of 2, in addition to the eight copy number-amplified fastq files, was aligned to chromosome 1 of GRCh37 with bwa-mem (v0.6) with the default options. The resulting SAM files were converted to BAM format with samtools (v1.15) using the default options. Finally, we ran the EHdn profile command (v0.9.0) with the minimum anchor mapping quality set to 50 and maximum IRR mapping quality set to 40. Finally, the Anchored IRR values were extracted by overlapping the STR coordinates with the de novo repeat expansion calls.

### Short-read and long-read DNA sequencing

We sequenced the Caki-1 and 786-O cell lines with both short-read sequencing (60× sequencing coverage, 150-bp paired-end sequencing on a NovaSeq 6000 instrument) and long-read sequencing (50× sequencing coverage, PacBio HiFi sequencing on a Sequel IIe instrument). We aligned the long reads to GRCh37 with pbmm2 (v1.7.0), using the parameters --sort --min-concordance-perc 70.0 --min-length 50. We aligned the short reads to GRCh37 with Sentieon (v202112.01) using parameters -K 10000000 -M, an implementation of BWA-MEM, and analysed the samples with EHdn, as described above. We included loci for which at least one sample had an Anchored IRR value of >0 for further analysis. Anchored IRR values >0 arise when the repeat length exceeds the sequencing read length. To benchmark EHdn against long-read sequencing data, we manually determined the TR length of a given locus in the long-read sequencing data. If the TR length in the long-read sequencing data exceeded the short-read sequencing read length of 150 bp, we considered that locus to have been confirmed.

The PacBio HiFi data were aligned to GRCh37 with pbmm2 (v1.7.0) and visualized at the *UGT2B7* locus with Tandem Repeat Genotyper (v0.2.0; https://github.com/PacificBiosciences/trgt).

### Analysis of rRE loci

To determine whether rREs were associated with any human diseases, rREs were mapped to genes with GREAT (v4.0.4, default settings)^52^. The resulting genes were analysed with Enrichr using Jensen Diseases^53^. The output of this analysis is available in Supplementary Table 4. To determine whether repeat expansions were associated with MSI-high cancers, we obtained data from ref. ^3^. The percentage of MSI-high cancers was obtained for colon adenocarcinoma (COAD), stomach adenocarcinoma (STAD), kidney renal cell carcinoma (KIRC), ovarian serous cystadenocarcinoma (OV), prostate adenocarcinoma (PRAD), head and neck squamous cell carcinoma (HNSC), liver hepatocellular carcinoma (LIHC), bladder urothelial carcinoma (BLCA), glioblastoma multiforme (GBM), skin cutaneous melanoma (SKCM), thyroid carcinoma (THCA) and breast invasive carcinoma (BRCA) and compared with the number of repeat expansions and the percentage of patients with at least one repeat expansion in the corresponding cancer type from the PCAWG dataset. We also overlapped cancer genomes containing rREs with the microsatellite mutation rate (data available for all but 157 PCAWG genomes analysed in this study), which we term the STR mutation rate, and MSI calls from ref. ^28^. The association of rREs with STR mutation rate was assessed with the two-tailed Wilcoxon rank-sum test. The association of rREs with MSI calls was assessed by chi-squared test with Yates’ correction.

To determine whether rREs were associated with known mutational signatures, we downloaded mutational signatures from the ICGC Data Coordination Center (DCC; https://dcc.icgc.org/releases/PCAWG/mutational_signatures/Signatures_in_Samples). We performed multiple linear regression for each SBS and DBS signature to identify predictors of the number of rREs present in a sample. To choose the predictors, we performed best subset selection on DBS and SBS signatures and included age as a possible confounding factor. We used statsmodels (v0.12.2) in Python and, specifically, the ordinary least-squares model found in the statsmodels.api.OLS module to estimate the coefficients of the selected predictors in their corresponding multiple linear regression model^54^.

To determine whether repeat expansions were associated with a difference in cytotoxic activity, we calculated cytotoxic activity as previously described for four cancers that had matching RNA-seq and WGS data^40^. For each locus, we compared the cytolytic activity for patients with a repeat expansion to that for patients without a detected repeat expansion using a Welch’s *t* test (a two-tailed test) with correction for multiple-hypothesis testing (Benjamini–Hochberg FDR *q* < 0.05). rREs were annotated with genic elements using annotatr (v1.18.1)^32^.

To determine whether rREs were associated with regulatory elements, we downloaded cCREs^33^ and mapped them to GRCh37 with LiftOver (UCSC) (*n* = 950,091 after removing 174 outliers)^55^. We determined the distance between rREs and cCREs with the bedtools closest command (v2.27.1)^56^ and compared this distance to that for a simple repeats catalogue^57^. To compare the distance to ENCODE cCREs, a Welch’s *t* test was performed.

To determine whether prostate cancer rREs were associated with prostate cancer susceptibility loci^35^, we calculated the distance to three sets of loci using the ‘bedtools closest’ command. We calculated the distance between (1) rREs present in prostate cancer samples and prostate cancer susceptibility loci, (2) rREs not present in prostate cancer samples and prostate cancer susceptibility loci and (3) simple repeats and prostate cancer susceptibility loci. To compare the distances between these three associations, we performed a Welch’s *t* test with FDR correction (Benjamini–Hochberg).

To determine whether rREs were associated with replication timing, we downloaded Repli-seq replication timing data for seven cell lines from the ENCODE website (NCI-H460, T470, A549, Caki2, G401, LNCaP and SKNMC)^58^. We selected regions for which all cell lines had concordant signals for analysis (early or late replication designations in agreement for each cell line at a given locus). We determined whether there was a difference in the distribution of rREs across early- and late-replicating regions compared with the simple repeats catalogue by using bootstrapping (*n* *=* 10,000). We sampled 54 loci (the number of rREs present in a concordant replication region) from rREs and simple repeats. A Welch’s *t* test was performed on the bootstrapped samples to estimate a *P* value. We applied FDR correction (Benjamini–Hochberg) to the estimated *P* values. To determine whether rRE status in *UGT2B7* was associated with survival outcome in patients with clear cell RCC (TCGA abbreviation, KIRC), we used Welch’s *t*-test quartile.

To identify motifs enriched and depleted in the rRE catalogue, we followed the same method as in the motifscan Python module (v1.3.0)^59^. We compared our rRE catalogue to the simple repeats catalogue (TRF) as a control. For each unique motif present, we built a contingency table specifying the count of rREs and simple repeats with and without the motif. Two one-tailed Fisher’s exact tests were applied to the table to test for significance in both directions, that is, enrichment and depletion. The ‘stats’ module in the Scipy Python package (v1.7.0) was used to conduct the significance test. Because multiple-hypothesis tests were performed, we applied FDR correction (Benjamini–Hochberg) for multiple-hypothesis testing to the *P* values, with a cut-off (FDR) of 0.01.

For the comparison of SNVs in COSMIC genes to rREs, we first divided the cancer genomes into two categories: an rRE cohort and a non-rRE cohort. The rRE cohort contained all genomes that had at least one rRE detected (*n* = 615), and the non-rRE cohort contained all genomes that had no rREs detected (*n* = 1,897). We then looked at the number of donors in the rRE cohort that had at least one mutation in a given gene (COSMIC tier 1 genes) *i* and the number of donors in the non-rRE cohort that had at least one mutation in a given gene *i* with a contingency table. We calculated the *P* value (Fisher’s exact test) for the significance of associating genes with either the rRE or non-rRE cohort. This *P*-value calculation was repeated for all COSMIC genes, using FDR at a significance level of 0.05 (Benjamini–Hochberg) to correct for multiple-hypothesis testing.

### Estimation of expansions in the general population

To estimate the frequency of rREs in the general population, EHdn (v0.9.0) was run on 1000 Genomes Project samples^60^ (*n* = 2,504) (GRCh38) and Medical Genome Reference Bank^61^ samples (*n* = 4,010) (GRCh37 lifted over to GRCh38).

The genomic coordinates of the 160 rREs (GRCh37) were padded with 1,000 bp and translated to GRCh38 coordinates with UCSC LiftOver. Then, the rRE coordinates (GRCh38) were overlapped with loci from the population samples containing Anchored IRR calls. rREs that overlapped with matching motifs in the population samples were selected for further analysis. We next sought to identify expanded rREs in the population samples to quantify their prevalence. To do so, we converted their global-normalized Anchored IRR values to be comparable to ICGC values. This step was necessary because sequencing read lengths in the PCAWG dataset are generally 100 bp while the read lengths in the 1000 Genomes and Medical Genome Reference Bank datasets are 150 bp. Conversion followed the formula (Anchored IRR, 100 bp) = 0.5 + 1.5 × (Anchored IRR, 150 bp)^16^. A sample in the population samples was counted as expanded if its Anchored IRR value was greater than the 99th percentile of Anchored IRR values in the normal samples from the PCAWG dataset, a threshold that is comparable to the threshold used to call expansions in tumour samples (Extended Data Fig. 4). In future rRE catalogues, for the rare instance where the estimated frequency of repeat expansions in the population samples is higher than expected, these data could be used to further filter rREs to improve the detection of cancer-specific repeat expansions.

To compare the length of TRs in normal samples with and without a matching rRE in a tumour sample, donors in the Prost-AdenoCA and Kidney-RCC cohorts whose data are available for download through the Cancer Collaboratory were included (*n* = 253). We used ExpansionHunter (v5.0.0) with the default options to genotype prostate and kidney cancer rREs in the normal samples of the selected donors. When there were two alleles of an rRE in a sample, both alleles were included and treated as distinct data points. For each rRE, we tested whether the distribution of genotypes from donors who had an expansion in their tumour samples differed from that for donors who did not have an expansion. Student’s *t* test was used to compute *P* values with FDR correction (Benjamini–Hochberg) to adjust for multiple-hypothesis testing.

### Association of rREs with gene expression

Matching RNA-seq and WGS data were available for Kidney–RCC, Ovary–AdenoCA, Panc–AdenoCA and Panc–Endocrine. RNA-seq data from these samples were obtained from the DCC (https://dcc.icgc.org/), and values were converted to transcripts per million (TPM). Normalized gene expression (TPM) values were compared for samples with and without an rRE (Welch’s *t* test, with FDR correction). For isoform analysis, normalized gene expression counts were compared for samples with and without a repeat expansion using the DESeq2 (v1.32.0) package in R (v4.0.5). We used the DESeq function to calculate the log_2_-transformed fold change for three isoforms of the *UGT2B7* gene (ENST00000305231.7, ENST00000508661.1 and ENST00000502942.1) and performed a Wald test with FDR correction using the Benjamini–Hochberg procedure (*q*-value threshold of *q* < 0.01).

### Design, synthesis and characterization of Syn-TEFs and polyamides

Syn-TEFs and polyamides were designed to target a GAAA repeat (Syn-TEF3 and PA3) or a control GGAA repeat (Syn-TEF4 and PA4). Syn-TEF3, Syn-TEF4, PA3 and PA4 were synthesized and purified to a minimum of 95% compound purity by WuXi Apptec and used without further characterization. HPLC conditions for chemical characterization were as follows: flow rate of 1.0 ml min^–1^; solvent A: 0.1% (vol/vol) trifluoroacetic acid (TFA) in water; solvent B: 0.075% (vol/vol) TFA in acetonitrile; Gemini column: C18 5 μm 110A 150 × 4.6 mm. Full results of characterization can be found in Supplementary Fig. 2.

### Treatment of RCC cell lines with Syn-TEFs

Caki-1, 786-O and Caki-2 cells were obtained from the American Type Culture Collection (ATCC) and grown in RPMI-1640 with l-glutamine (Gibco, 11875093), supplemented with 10% (vol/vol) FBS. A498 and ACHN cells were obtained from ATCC and grown in DMEM with glucose, l-glutamine and sodium pyruvate (Corning, 10-013-CV), supplemented with 10% (vol/vol) FBS. RCC-4 cells were obtained from A. Giacca (Stanford University) and grown in DMEM with glucose, l-glutamine and sodium pyruvate (Corning, 10-013-CV), supplemented with 10% (vol/vol) FBS. Cell line identities were confirmed by STR profiling (Genetic Resource Core Facility, Johns Hopkins University) and tested negative for mycoplasma. Cells were seeded in 96-well plates on day 0. On day 1, cells were treated with the indicated molecules. Molecules were dissolved in DMSO (vehicle) and added to cells (0.1% (vol/vol) final concentration of DMSO). On day 4 (72 h later), relative metabolic activity was measured as a proxy for relative cell density, using the Cell Counting Kit (CCK-8, Dojindo Molecular Technologies) according to the manufacturer’s instructions. Absorbance (450 nm) of cells treated with molecules was normalized to that for cells treated with DMSO (0.1% (vol/vol)) or with no treatment. Absorbance was measured with an Infinite M1000 microplate reader (Tecan).

For microscopy, Caki-1 and 786-O cells were plated on glass-bottom 96-well plates under standard culture conditions. One day after plating, medium containing no drug, 50 μM Syn-TEF3 or 50 μM Syn-TEF4 was added, and the cells were incubated for 72 h at 37 °C. As a control, wells that received no treatment were incubated with 70% (vol/vol) ethanol for 30 s before staining. Cells were then stained with propidium iodide, Calcein-AM and Hoechst 33342 from the Live-Dead Cell Viability Assay kit (Millipore Sigma, CBA415) according to the manufacturer’s instructions and immediately imaged at ×10 magnification with a 0.17-NA CFI60 objective on a Keyence BZ-X710 microscope. Eight fields were measured for each treatment condition, and the experiment was repeated two times. Quantification was conducted using FIJI software (release 20220330-1517). For statistical analyses, one-way ANOVA adjusted with Bonferroni correction for multiple comparisons was conducted with GraphPad Prism (v9.3.1).

### Statistics and reproducibility

Data are represented as the mean ± s.e.m. unless stated otherwise. All experiments were reproduced at least twice unless stated otherwise. Box plots were prepared with matplotlib (v3.4 or v3.6) as follows unless stated otherwise: the box extends from the first quartile (Q1 or 25th percentile) to the third quartile (Q3 or 75th percentile) of the data, with a line at the median. The whiskers extend from the box by 1.5 times the interquartile range (IQR). The IQR is the difference between the values at Q3 and Q1. Outliers were not plotted to improve clarity. Details on how box plots were generated are available at https://matplotlib.org/stable/api/_as_gen/matplotlib.axes.Axes.boxplot.html#matplotlib.axes.Axes.boxplot.

### Reporting summary

Further information on research design is available in the Nature Portfolio Reporting Summary linked to this article.

## Online content

Any methods, additional references, Nature Portfolio reporting summaries, source data, extended data, supplementary information, acknowledgements, peer review information; details of author contributions and competing interests; and statements of data and code availability are available at 10.1038/s41586-022-05515-1.

## Supplementary information

Supplementary Information This file contains Supplementary Figs. 1 and 2.

Reporting Summary

Supplementary Tables This file contains Supplementary Tables 1–7.

## Data Availability

Access to the PCAWG dataset can be obtained by applying for access at https://daco.icgc.org/. WGS data (both short- and long-read DNA sequencing) for the 786-O and Caki-1 cell lines have been deposited in NCBI with accession PRJNA868795.

## Code availability

The code to filter loci from EHdn, termed TROPIC, is available on GitHub at https://github.com/AshwiniS7/TROPIC-Tandem-Repeat-Locus-Prioritization-in-Cancer. The code to perform local read depth normalization is available on GitHub at https://github.com/rashidalabri/cancer-rre-paper-analysis.
