# Supplementary material for: Recurrent repeat expansions in human cancer genomes
Source: Nature. 2022 Dec 14;613(7942):96–102. doi: 10.1038/s41586-022-05515-1 (PMC9812771; doi:10.1038/s41586-022-05515-1)
Supplement: Supplementary file 1 — This file contains Supplementary Figs. 1 and 2. [file 41586_2022_5515_MOESM1_ESM.pdf]

nature portfolio
https://doi.org/10.1038/s41586-022-05515-1

Supplementary information

Recurrent repeat expansions in human cancer genomes

In the format provided by the authors and unedited

Guide to Supplementary Information

Supplementary Figure 1

Unprocessed gels associated with the data in Figure 4a and Extended Data Figure 8a.

Supplementary Figure 2

Details of polyamide and Syn-TEF structure, synthesis, and characterization.

Supplementary Tables (xlsx file)

Table 1: Catalog of rREs (v1.0)

Table 2: List of samples from PCAWG used in this study

Table 3: Results of filtering TRs to identify rREs

Table 4: Underlying data from Fig. 3a describing association with Jensen diseases. P-value from one-tailed Fisher's exact test, q-values were prepared with Benjamini-Hochberg to correct for multiple hypothesis testing.

Table 5: Primers used for analysis of TRs

Table 6: Estimation of likelihood of detecting rREs in the independent cohorts of samples studied

Table 7: Comparison of the genotype of normal samples from patients with tumors containing an rRE compared to the genotype of normal samples from patients with tumors containing no rRE. P-value from one-tailed Fisher's exact test, q-values were prepared with Benjamini-Hochberg to correct for multiple hypothesis testing.

Source gel for Fig. 4a

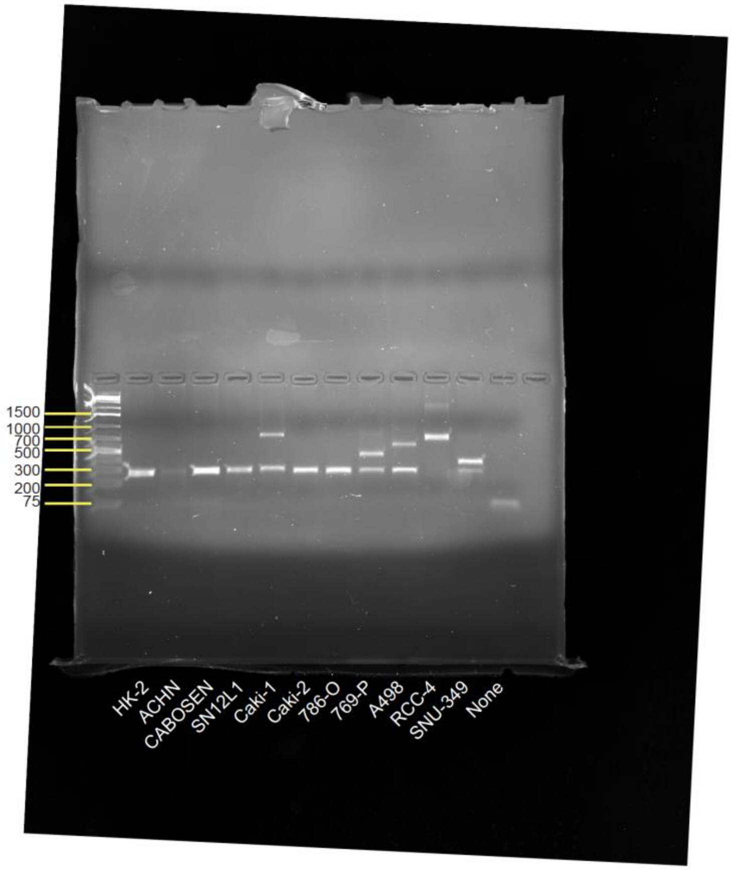

Source gel for Extended Data Fig. 8a. N, Normal tissue; T, tumor tissue

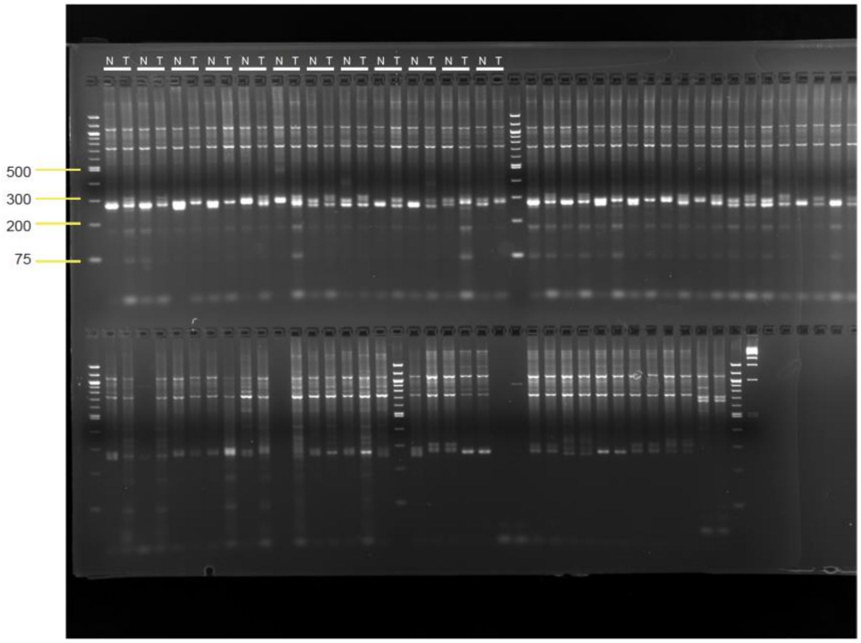

Figure S1. Unprocessed gels associated with the data in Figure 4a and Extended Data Figure 8a. Units for the ladder are base pairs (bp).

a

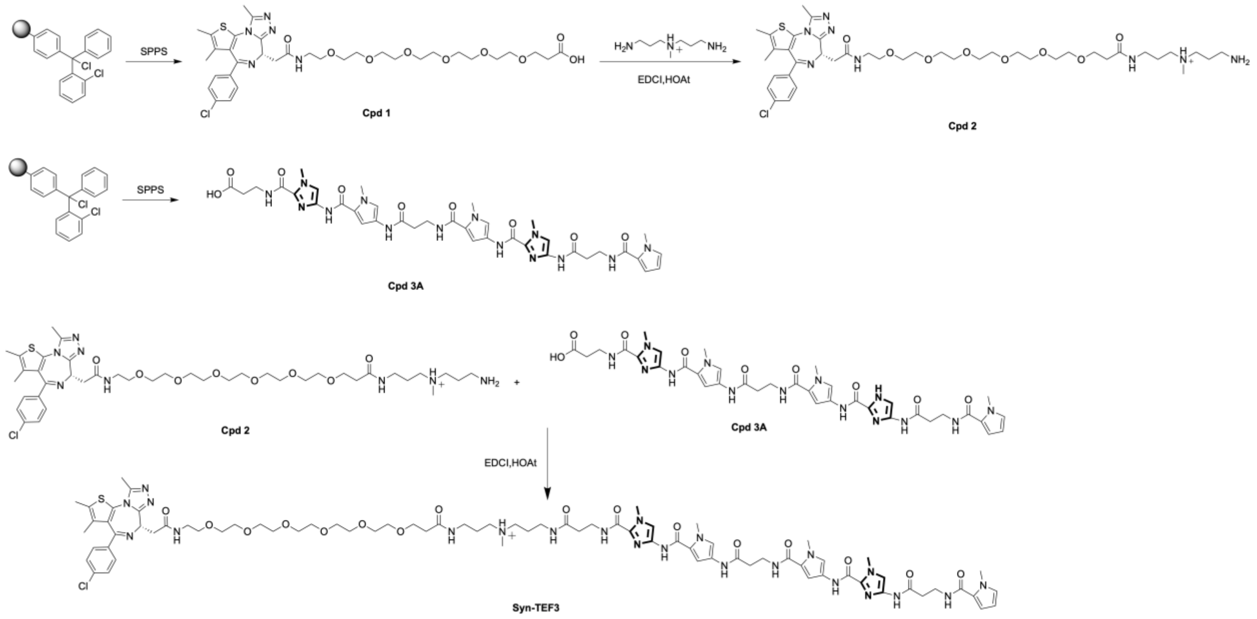

b

Syn-TEF3

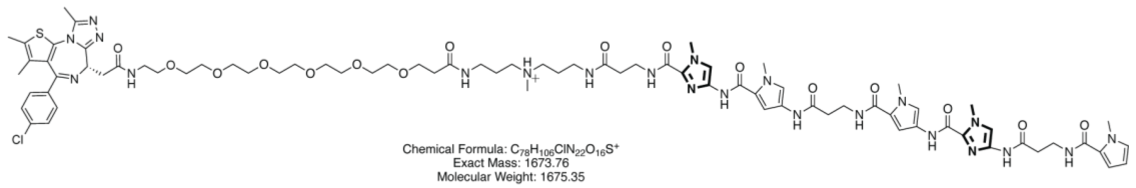

HPLC

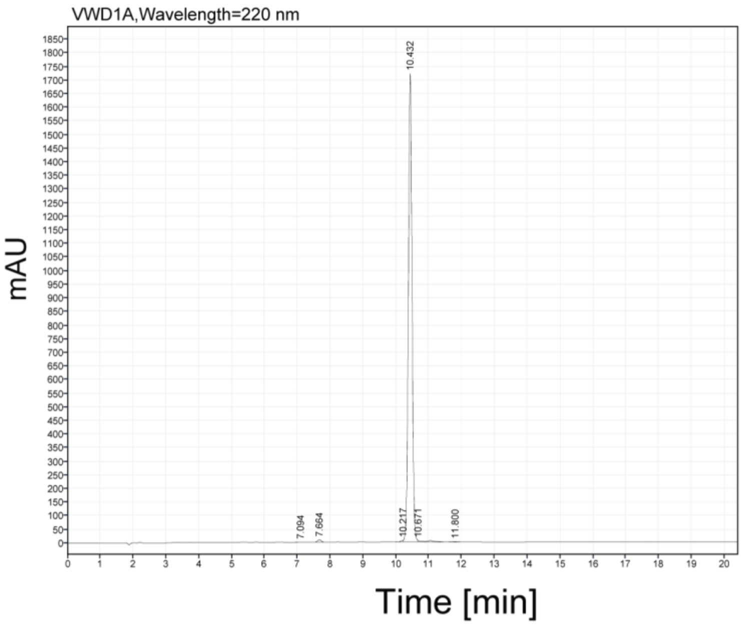

LC-MS

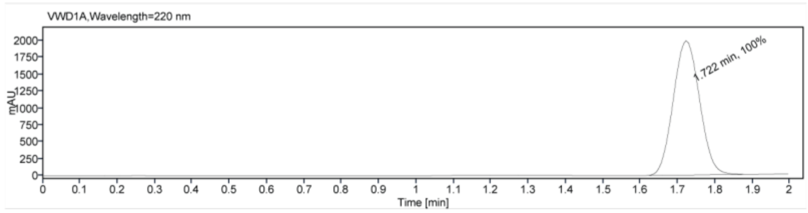

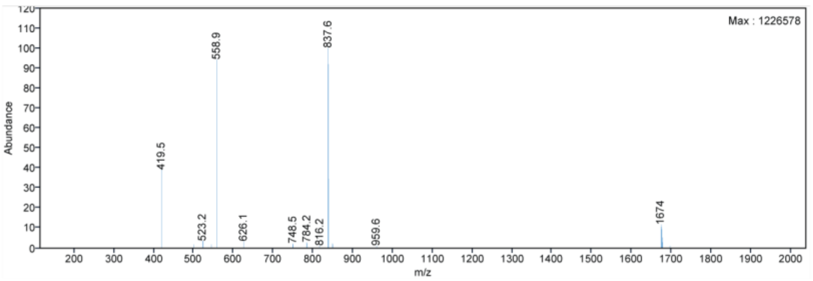

c

Syn-TEF4

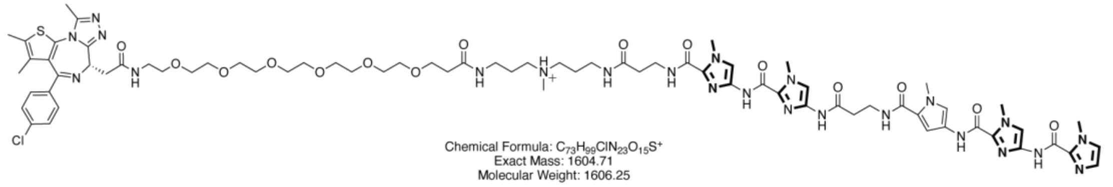

Chemical Formula: $C_{73}H_{99}ClN_{23}O_{15}S^+$
Exact Mass: 1604.71
Molecular Weight: 1606.25

HPLC

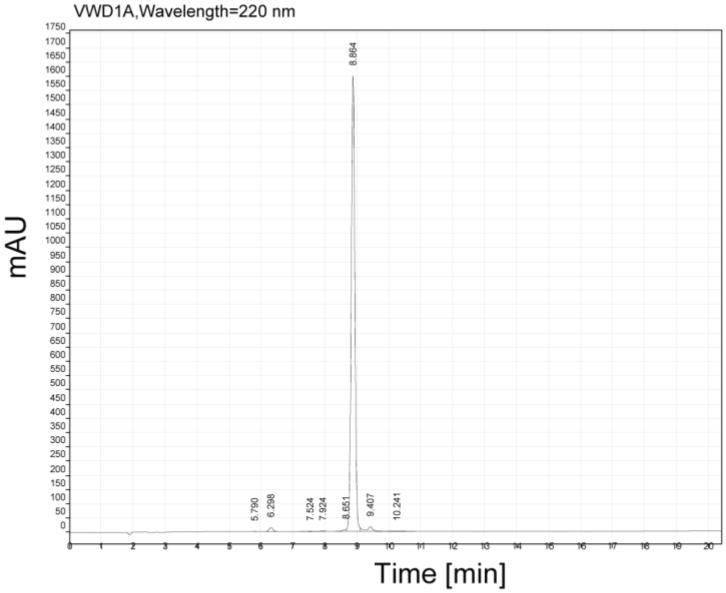

LC-MS

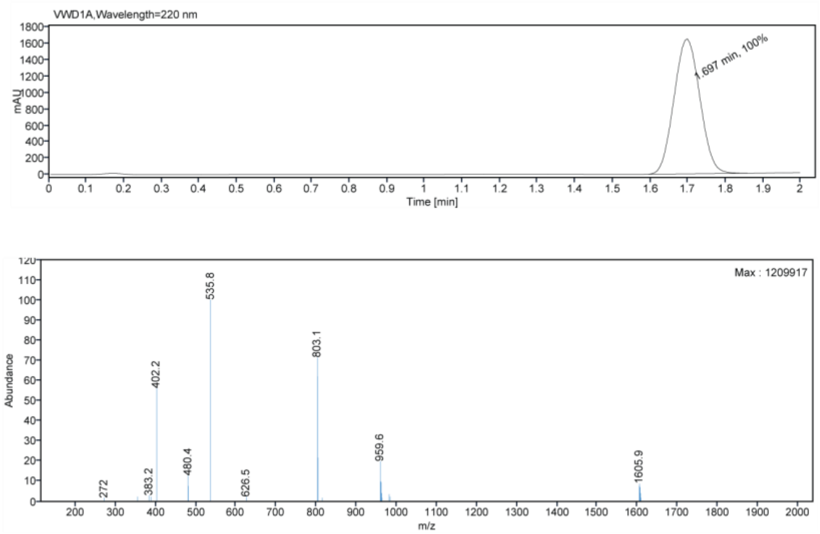

d

PA3

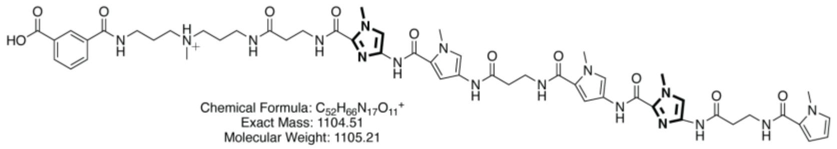

HPLC

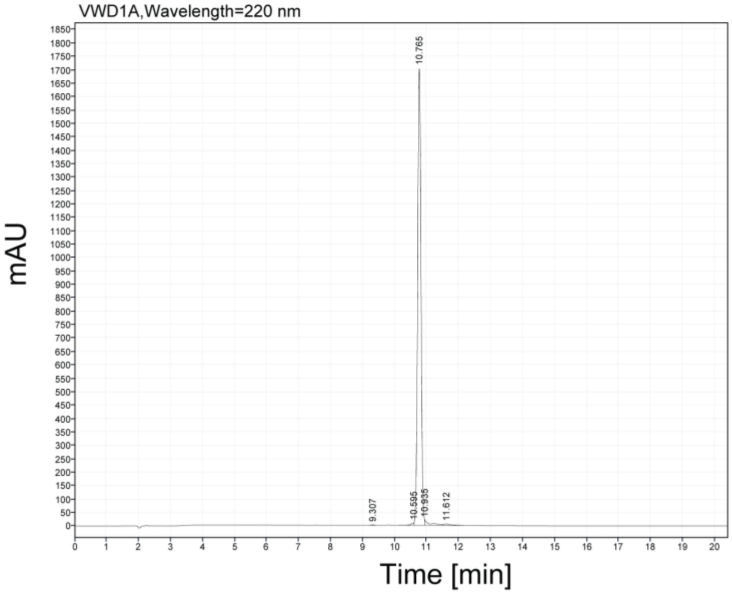

LC-MS

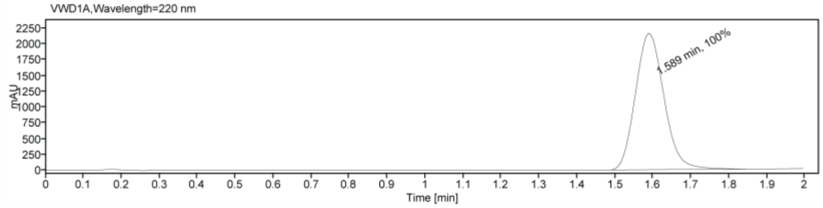

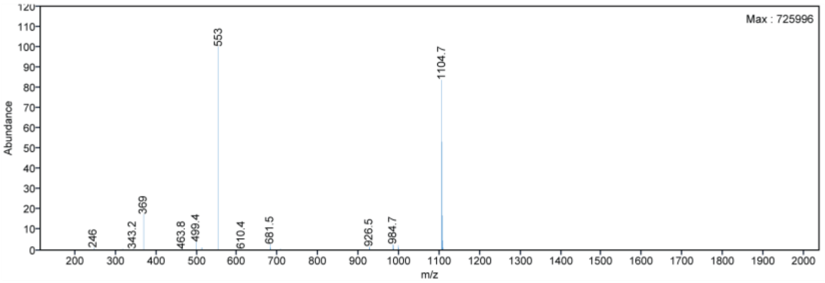

e

PA4

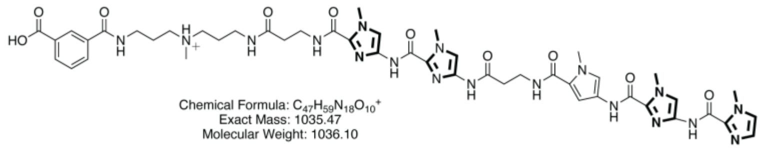

HPLC

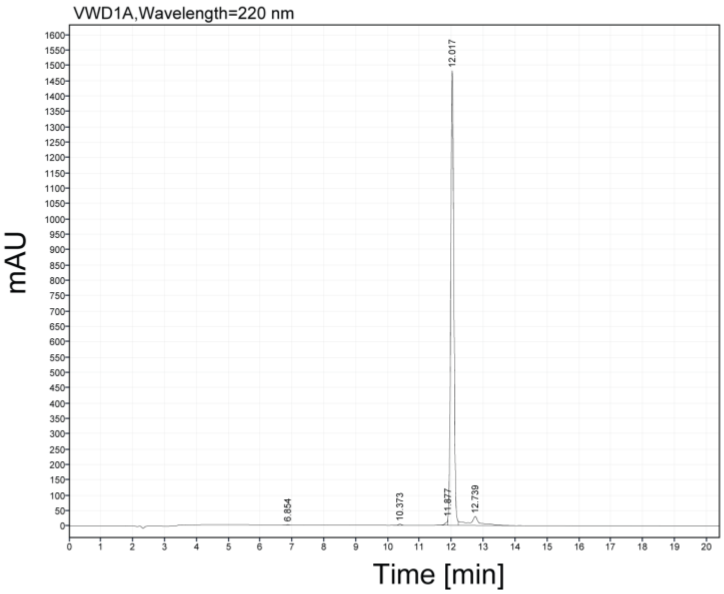

LC-MS

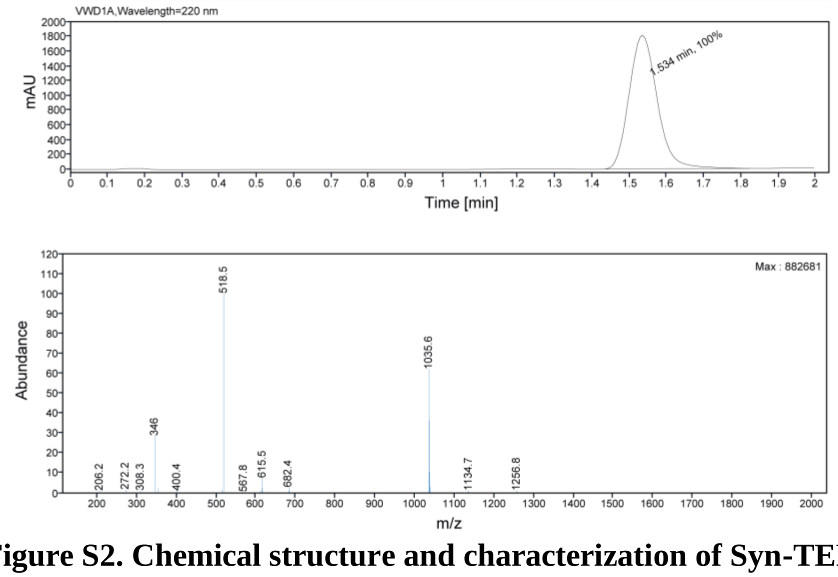

Figure S2. Chemical structure and characterization of Syn-TEFs and PAs. a) Representative synthetic scheme for Syn-TEF3. *N*-methylimidazole is bolded for clarity. Chemical structure and characterization by HPLC and LC-MS of b) Syn-TEF3, c) Syn-TEF4, d) PA3, and e) PA4.
