# Supplementary material for: Recurrent repeat expansions in human cancer genomes
Source: Nature. 2022 Dec 14;613(7942):96–102. doi: 10.1038/s41586-022-05515-1 (PMC9812771; doi:10.1038/s41586-022-05515-1)
Supplement: Supplementary file 2 — Reporting Summary [file 41586_2022_5515_MOESM2_ESM.pdf]

nature portfolio

Corresponding author(s): Graham Erwin, Michael Snyder, Mark Gerstein

Last updated by author(s): Oct 22, 2022

Reporting Summary

Nature Portfolio wishes to improve the reproducibility of the work that we publish. This form provides structure for consistency and transparency in reporting. For further information on Nature Portfolio policies, see our Editorial Policies and the Editorial Policy Checklist.

Statistics

For all statistical analyses, confirm that the following items are present in the figure legend, table legend, main text, or Methods section.

n/a	Confirmed	
☐	☒	The exact sample size (*n*) for each experimental group/condition, given as a discrete number and unit of measurement
☐	☒	A statement on whether measurements were taken from distinct samples or whether the same sample was measured repeatedly
☐	☒	The statistical test(s) used AND whether they are one- or two-sided<br>*Only common tests should be described solely by name; describe more complex techniques in the Methods section.*
☐	☒	A description of all covariates tested
☐	☒	A description of any assumptions or corrections, such as tests of normality and adjustment for multiple comparisons
☐	☒	A full description of the statistical parameters including central tendency (e.g. means) or other basic estimates (e.g. regression coefficient) AND variation (e.g. standard deviation) or associated estimates of uncertainty (e.g. confidence intervals)
☐	☒	For null hypothesis testing, the test statistic (e.g. *F*, *t*, *r*) with confidence intervals, effect sizes, degrees of freedom and *P* value noted<br>*Give P values as exact values whenever suitable.*
☒	☐	For Bayesian analysis, information on the choice of priors and Markov chain Monte Carlo settings
☒	☐	For hierarchical and complex designs, identification of the appropriate level for tests and full reporting of outcomes
☒	☐	Estimates of effect sizes (e.g. Cohen's *d*, Pearson's *r*), indicating how they were calculated

Our web collection on statistics for biologists contains articles on many of the points above.

Software and code

Policy information about availability of computer code

Data collection	No software was used for data collection.
Data analysis	R v4.0.5<br>Python v3.9.6<br>BioPython v1.79<br>wgsim v0.3.1-r13<br>ExpansionHunter v4.0.2 and v5.0.0<br>ExpansionHunter De Novo v0.9.0<br>GREAT v4.0.4<br>REViewer v0.1.1<br>Annotatr v1.18.1<br>samtools v1.13 and v1.15<br>bedtools v2.27.1<br>Python scipy library v1.3.1 and v1.7.0<br>statsmodels v0.12.2<br>DESeq2 v1.32.0<br>Sentieon v202112.01<br>pbmm2 v1.7.0<br>Tandem Repeat Genotyper v0.2.0<br>GREAT v4.0.4<br>statsmodel v0.12.2

March 2021

motifscan v1.3.0
GraphPad Prism v9.3.1
Fiji (release 20220330-1517)
matplotlib v3.4 and v3.6

For manuscripts utilizing custom algorithms or software that are central to the research but not yet described in published literature, software must be made available to editors and reviewers. We strongly encourage code deposition in a community repository (e.g. GitHub). See the Nature Portfolio guidelines for submitting code & software for further information.

Data

Policy information about availability of data

All manuscripts must include a data availability statement. This statement should provide the following information, where applicable:

- Accession codes, unique identifiers, or web links for publicly available datasets
- A description of any restrictions on data availability
- For clinical datasets or third party data, please ensure that the statement adheres to our policy

Access to the PCAWG dataset can be obtained by applying for access: https://daco.icgc.org/
Whole-genome sequencing data (both short- and long-read DNA sequencing) from 786-O and Caki-1 cell lines are deposited in NCBI with accession PRJNA868795.

Human research participants

Policy information about studies involving human research participants and Sex and Gender in Research.

Reporting on sex and gender	Sex and gender were not collected for matching normal tumor pair samples in this study. However, we note that prostate and breast cancer overwhelmingly affect men and women, respectively.
Population characteristics	Samples are from patients diagnosed with prostate cancer, breast cancer, or kidney cancer.
Recruitment	Patients were recruited by their oncologists. Not all patients who were approached decided to participate in the study, hence there is potential self-selection bias, though the authors consider the possibility unlikely that this translates to a bias in any biologic variable. Similarly, as this is a single-institution study, the racial and ethnic distribution in the study cohort represents the distribution in the catchment area of the Stanford Medical Center, which is different from the general population, and therefore constitutes a potential bias.
Ethics oversight	The collection of samples from human research participants used in this study was approved by the IRB at Stanford University.

Note that full information on the approval of the study protocol must also be provided in the manuscript.

Field-specific reporting

Please select the one below that is the best fit for your research. If you are not sure, read the appropriate sections before making your selection.

☒ Life sciences ☐ Behavioural & social sciences ☐ Ecological, evolutionary & environmental sciences

For a reference copy of the document with all sections, see nature.com/documents/nr-reporting-summary-flat.pdf

Life sciences study design

All studies must disclose on these points even when the disclosure is negative.

Sample size	We obtained the maximum possible number of whole-genome sequence data available to us from PCAWG, so no statistical methods were used to predetermine the sample size for this analysis. However, based on previously published work in the field that have performed similar analyses we reasoned that the dataset from PCAWG was sufficient our studies here.
Data exclusions	We included all white-listed whole-genome sequencing datasets that we could obtain from the PCAWG consortium. Note, the term "white-listed" is from the PCAWG Consortium and refers to samples that passed a quality control check; this term is explained in the Methods section of the manuscript. We analyzed cancers with at least 20 matching tumor-normal pairs. This step was pre-determined.
Replication	No replication cohort with similar size of whole-genome sequencing data exists for these data.
Randomization	Randomization is not relevant because WGS samples from PCAWG are not assigned to an experimental group for this analysis. No randomization was performed.
Blinding	Blinding is not relevant because WGS samples from PCAWG are not assigned to an experimental group for this analysis. No blinding was performed.

Reporting for specific materials, systems and methods

We require information from authors about some types of materials, experimental systems and methods used in many studies. Here, indicate whether each material, system or method listed is relevant to your study. If you are not sure if a list item applies to your research, read the appropriate section before selecting a response.

Materials & experimental systems

n/a	Involved in the study
☒	☐ Antibodies
☐	☒ Eukaryotic cell lines
☒	☐ Palaeontology and archaeology
☒	☐ Animals and other organisms
☒	☐ Clinical data
☒	☐ Dual use research of concern

Methods

n/a	Involved in the study
☒	☐ ChIP-seq
☒	☐ Flow cytometry
☒	☐ MRI-based neuroimaging

Eukaryotic cell lines

Policy information about cell lines and Sex and Gender in Research

Cell line source(s)	Caki-1, Caki-2, ACHN, A498, and 786-O cell lines originated from ATCC. RCC-4 was from the lab of Amato Giacca, Stanford University
Authentication	Cell line identities were authenticated by STR profiling by the Genetic Resources Core Facility at Johns Hopkins University, with the exception of SNU-349, which did not match the reported STR profile of SNU-349 or any other catalogued cell line, but has a mutated VHL gene and expresses high levels of PAX8 and CA9, consistent with ccRCC origin.
Mycoplasma contamination	All cell lines tested negative for mycoplasma contamination with the MycoAlert Mycoplasma Detection Kit (Lonza)
Commonly misidentified lines (See ICLAC register)	No commonly misidentified cell lines were used in this study.
